# Up-Scalable Fabrication of SnO_2_ with Multifunctional Interface for High Performance Perovskite Solar Modules

**DOI:** 10.1007/s40820-021-00675-7

**Published:** 2021-07-10

**Authors:** Guoqing Tong, Luis K. Ono, Yuqiang Liu, Hui Zhang, Tongle Bu, Yabing Qi

**Affiliations:** Energy Materials and Surface Sciences Unit (EMSSU), Okinawa Institute of Science and Technology Graduate University (OIST), 1919-1 Tancha, Onna-son, Kunigami-gun, Okinawa, 904-0495 Japan

**Keywords:** Perovskites, Solar modules, Operational stability, Interface passivation, SnO_2_

## Abstract

Tin dioxide (SnO_2_) has been demonstrated as one of the promising electron transport layers for high-efficiency perovskite solar cells (PSCs). However, scalable fabrication of SnO_2_ films with uniform coverage, desirable thickness and a low defect density in perovskite solar modules (PSMs) is still challenging. Here, we report preparation of high-quality large-area SnO_2_ films by chemical bath deposition (CBD) with the addition of KMnO_4_. The strong oxidizing nature of KMnO_4_ promotes the conversion from Sn(II) to Sn(VI), leading to reduced trap defects and a higher carrier mobility of SnO_2_. In addition, K ions diffuse into the perovskite film resulting in larger grain sizes, passivated grain boundaries, and reduced hysteresis of PSCs. Furthermore, Mn ion doping improves both the crystallinity and the phase stability of the perovskite film. Such a multifunctional interface engineering strategy enabled us to achieve a power conversion efficiency (PCE) of 21.70% with less hysteresis for lab-scale PSCs. Using this method, we also fabricated 5 × 5 and 10 × 10 cm^2^ PSMs, which showed PCEs of 15.62% and 11.80% (active area PCEs are 17.26% and 13.72%), respectively. For the encapsulated 5 × 5 cm^2^ PSM, we obtained a T_80_ operation lifetime (the lifespan during which the solar module PCE drops to 80% of its initial value) exceeding 1000 h in ambient condition.

**Supplementary Information:**

The online version contains supplementary material available at 10.1007/s40820-021-00675-7.

## Highlights

- High-quality large-area SnO_2_ films are fabricated by chemical bath deposition with the addition of KMnO_4_.
- The presence of K and Mn ions can improve both the crystallinity and the phase stability of perovskites and reduce hysteresis of devices simultaneously.
- Perovskite solar modules (22.4 and 91.8 cm^2^) are demonstrated with active area efficiencies of 17.26% and 13.72%, respectively, and the 22.4 cm^2^ perovskite solar module exhibits a *T*_80_ operation lifetime exceeding 1000 h in ambient condition.

**Supplementary Information:**

The online version contains supplementary material available at 10.1007/s40820-021-00675-7.

## Graphical abstract

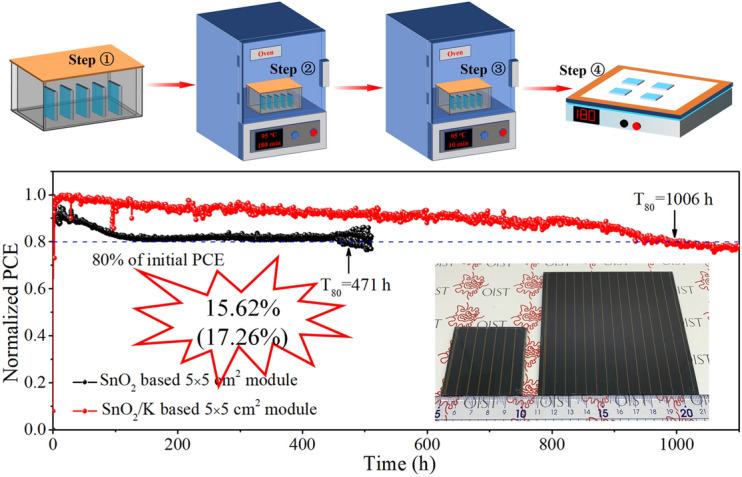

## Introduction

The attributes of high performance, simple fabrication process and low-cost make perovskite solar cells (PSCs) a good candidate in the field of energy conversion [1–7]. The record powder conversion efficiency (PCE) of 25.5% for lab-scale PSCs now is comparable with the commercial photovoltaics [8, 9]. Up to now, the high performance of PSCs is mainly based on a planar or mesoporous structure [10–14]. In these architectures, the electron transport layer (ETL) as the bottom layer is the key in the device fabrication toward high efficiency and long-term stability [15]. Tin dioxide (SnO_2_) has been demonstrated as an efficient ETL candidate in the case of PSCs due to its excellent mobility, high light transmittance and suitable energy level alignments with perovskites [16–19]. It can be easily coated on the planar and/or tandem structure by spin-coating [12, 20, 21], chemical bath deposition (CBD) [22, 23], sputtering deposition [24] and atomic layer deposition (ALD) [25–27].

High-quality and advanced fabrication techniques of SnO_2_ films not only help achieve a high efficiency in lab-scale, but also promote the development of perovskite solar modules (PSMs) [28]. To date, the record mini-module efficiency of PSMs is over 18.6% with a module area of ~ 30 cm^2^ [29]. However, the stability still lags behind because of poor interface and fragile perovskite materials [20]. Therefore, high efficiency and long-term stability of PSMs not only need a high-quality perovskite active layer, but also require the preparation of ETLs and interface modification [20, 30, 31]. Better uniformity across the large area, simple fabrication process and low-cost/temperature of ETLs are imperative for the upscalable fabrication of PSMs [32]. However, when upscaling PSMs the risk to form voids/pinholes inevitably increases in the conventional spin-coating process because of the thickness fluctuation in the ultra-thin SnO_2_ layer [33]. Also, the mesoporous layer as ETL adds additional complexity in the module fabrication and increases the series resistance simultaneously [34, 35]. Thus, a compact, high crystalline and high mobility ETLs are important for module fabrication. Sputtering deposition has been commonly used to deposit uniform SnO_2_ thin films across a large area at low temperature; however, the low crystallization and the large density of defects lead to poor carrier extraction [24]. The relatively high-cost of ALD makes it less desirable for mass production [17]. Although conventional CBD is a low-cost process compatible with upscalable deposition, the need of a seed layer and additional surface passivation make the deposition process complex [22]. Recently, CBD was demonstrated as an efficient way to produce high-quality SnO_2_ by precisely controlling the thickness, which led to a record output efficiency of 25.2% in lab-scale [36]. However, large area SnO_2_ deposition is still rarely reported using the CBD process (Table S1) because additional surface treatments are needed to passivate the oxygen vacancies and surface hydroxyl groups in the SnO_2_ film [20, 37–40], which not only increases the nonradiative recombination, but also accelerates the degradation of the devices[40, 41]. It is worth noting that the conventional surface passivation of SnO_2_ layer is mainly based on spin-coating techniques or additional spin-coating process [20, 37, 39–43], which makes them challenging for large-scale fabrication. Therefore, it is vital to achieve high-quality SnO_2_ thin films and proper SnO_2_/perovskite interfaces by a more facile approach.

In this work, we prepared the SnO_2_ thin layers on both lab-scale and upscalable substrates by introducing the potassium permanganate (KMnO_4_) into the precursor solution in the CBD method (abbreviated as SnO_2_/K-ETL; SnO_2_ without KMnO_4_ is abbreviated as SnO_2_-ETL). Strong oxidant of KMnO_4_ not only facilitates the complete oxidation of the SnCl_2_ precursor solution, but also provides additional treatment by K and Mn ions. K ions can diffuse into perovskites to enlarge grain size and passivate the grain boundaries (GBs) simultaneously. Unintentional Mn doping not only improves the crystallinity of perovskite films, but also enhances the phase stability of perovskites. Moreover, the presence of K ions can effectively reduce the hysteresis of PSCs/PSMs. This multifunctional interface engineering strategy led to a champion power conversion efficiency (PCE) of 21.70% for the SnO_2_/K-based PSCs (active area = 0.09 cm^2^). Furthermore, the large area PSMs of 5 × 5 and 10 × 10 cm^2^ were fabricated to generate the module PCEs of 15.62% (active area PCE = 17.26%) and 11.80% (active area PCE = 13.72%), respectively. A *T*_80_ lifetime (the lifespan during which the solar module PCE drops to 80% of its initial value) over 1000 h was demonstrated on the 5 × 5 cm^2^ PSM under continuous AM 1.5G light illumination at a fixed bias in ambient condition (relative humidity RH ~ 55%, 25 °C).

## Experimental Section

### Materials

Formamidinium iodide (FAI), methylammonium iodide (MAI), methylammonium bromide (MABr) and methylammonium chloride (MACl) were purchased from Greatcell solar (Australia). PbI_2_ (99.99%) was purchased from TCI. 2,2′,7,7′-tetrakis(N,N-di-p-methoxyphenyl-amine)9,9′-spirobifluorene (Spiro-OMeTAD) was purchased from Xi’an Polymer Light Technology (China). Dimethylformamide (DMF), dimethyl sulfoxide (DMSO), isopropanol (IPA), chlorobenzene (CB), and hydrochloric acid (HCl, 36.4%) were purchased from Wako (Japan). 4-tert-butylpyridine (99.9%), acetonitrile (99.9%), SnCl_2_·2H_2_O (99.99%), urea and mercaptoacetic acid (98%) were purchased from Sigma-Aldrich. Potassium permanganate (KMnO_4_, 99.3%) was purchased from Nacalai Tesque. All reagents were used as received without further purification.

### SnO_2_ Thin Film

The SnO_2_ thin film was deposited by CBD method. Specifically, 5 g urea was dissolved into 400 mL distilled water in a glassware. After stirring for 5 min, 100 µL mercaptoacetic acid, 5 mL HCl and 1.1 g SnCl_2_·2H_2_O were sequentially dropped into solution and kept stirring for another 5 min. The solution needs to be diluted four times before use. The cleaned fluorine-doped tin oxide (FTO) substrates were placed vertically in the glassware that contained SnCl_2_ precursor solution. Then, the glassware was placed in an oven with a temperature of 95 °C. After 3 h heating, the different amounts of KMnO_4_ were added into glassware. After 10 min continuous heating, the glassware was taken out followed by cleaning FTO substrates. Finally, the as-prepared FTO/SnO_2_ substrates were annealed at 180 °C for 1 h in ambient.

### Devices Fabrication

FTO substrates (1.5 × 1.5 cm^2^) were sequentially washed by the detergent, distilled water and IPA for 30 min, respectively. Then, the FTO substrates were dried by N_2_ flow and treated by using UV-ozone for one hour. After deposition of SnO_2_ by CBD method, the perovskite film (Cs_0.05_FA_0.85_MA_0.1_PbI_2.85_Br_0.15_) was fabricated by two-step spin-coating. First, 1.35 M PbI_2_ and 0.0675 M CsI were dissolved in mixed solution (1.9 mL DMF and 0.1 mL DMSO) and stirred at 70 °C for 30 min. The PbI_2_ precursor solution was coated on FTO/SnO_2_ substrates by spin-coating (3000 rpm, 30 s). Second, the mixed FAI/MAI/MABr/MACl precursor solution (FAI 1000 mg; MAI 110 g; MABr 110 mg; MACl 110 mg, dissolved in 15 mL IPA) was spin-coated (3000 rpm, 30 s) on the top of the FTO/SnO_2_/PbI_2_. To obtain the perovskite layer, the as-prepared films were annealed at 150 °C for 15 min in ambient environment (RH ~ 20%, 20 °C). 100 μL of spiro-OMeTAD solution (spiro-OMeTAD solution was prepared as reported in our previous works [20, 44].) was spin-coated on the perovskite film at 3500 rpm for 30 s. Finally, 100 nm gold electrode was coated by thermal evaporation under a high vacuum pressure of 1 × 10^−5^ Torr. Except for the gold evaporation, all the processes were performed in an ambient environment with a relative humidity (RH) of ~ 20%.

### PSM Fabrication

The FTO substrates of 5 × 5 and 10 × 10 cm^2^ PSMs were cleaned by the detergent, distilled water and IPA for 30 min, respectively. The SnO_2_ film, perovskite layer, spiro-OMeTAD film and gold electrode were deposited by CBD, spin-coating and thermal evaporation method, respectively, as we discussed before. For the 5 × 5 cm^2^ PSM, the FTO substrate contains 7 sub-cells with a defined size of 6.65 mm by 49 mm. Similarly, 14 sub-cells with a defined size of 6.65 mm by 99 mm were made in 10 × 10 cm^2^ PSM. The gap between each FTO pattern is named as P1 with a width of 50 μm. After deposition of SnO_2_ film, perovskite layer and spiro-OMeTAD, another line defined as P2 was carried out by laser scribing via a CO_2_ laser (output intensity is ~ 5.6 W) to expose the bottom FTO electrodes in order to construct the series connections between the sub-cells in the PSMs. Finally, P3 line was performed by mechanical scribing to sperate each sub-cell after gold evaporation. The laser scribing process and thermal evaporation were conducted in an ambient environment with a RH of ~ 55%. All the other processes were performed in a dry condition (RH ~ 20%).

### Photovoltaic Characterization

The *J–V* curves of the lab-scale PSCs (1.5 × 1.5 cm^2^) and PSMs (5 × 5 cm^2^) are carried out under AM 1.5 G, 100 mW cm^−2^ by using a solar simulator (Newport Oriel Sol 1A, Xenon-lamp, USHIO, UXL-150SO) and Keithley 2420 source meter. The *J–V* curves of 10 × 10 cm^2^ PSMs were measured under AM 1.5 G, 100 mW cm^−2^ by using a solar simulator with an output beam of 8 × 8 inches (Newport Oriel Sol 3A, Xenon-lamp, 94083A). All the solar simulators are calibrated by using a KG3 reference Si-cell (Enlitech, Oriel Instruments Model Number 90026564, 2 × 2 cm^2^) before test. The lab-scale PSCs were measured under reverse scan (1.3 to −0.1 V) and forward scan (−0.1 to 1.3 V) with a scan rate of 0.13 V s^−1^. Similarly, the J-V scan range of PSMs are from 12 to −0.2 V (forward: −0.2 to 12 V) and 16.0 to −0.2 V (forward: −0.2 to 16 V) for 5 × 5 cm^2^ PSMs and 10 × 10 cm^2^ PSMs, respectively. The scan rate for PSMs is 0.25 V s^−1^. No preconditioning protocol was used before the characterization in this work. The EQE spectra of lab-scale PSCs were characterized from 300 to 850 nm using Oriel IQE 200. The lab-scale PSCs (1.5 × 1.5 cm^2^) were tested by using a metal shadow mask with an aperture area of 0.09 cm^2^ and the designated area of 22.4 cm^2^ for 5 × 5 cm^2^ PSMs and 91.8 cm^2^ for 10 × 10 cm^2^ PSMs were defined by a corresponding metal mask.

### Characterization

XRD measurements (from 10° to 60°) were investigated by using a Bruker D8 Discover instrument (Bruker AXS GmbH, Karlsruhe, Germany) equipped with Cu wavelength *I* = 1.54 Å X-ray source operated at 1600 W and Goebel mirror. The scan interval is 0.02° per step. The top-view and cross section SEM images were investigated by using a scanning electron microscope (Helios NanoLab G3 UC, FEI). Surface investigations were performed by ultraviolet photoemission spectroscopy (UPS) and *X*-ray photoelectron spectroscopy (XPS). An *X*-ray photoelectron spectrometer (XPS-AXIS Ultra HAS, Kratos) equipped with monochromatic Al-Kα = 1486.6 eV and nonmonochromatic He-I = 21.22 eV sources was used for measurements. For sputtering in secondary ion mass spectroscopy (SIMS), a 3 keV Ar^+^ primary beam with a current of 10 mA and a diameter of 100 µm was used. The steady-state photoluminescence (PL) spectra were obtained by using a JASCO FP-8500 spectrometer. The time-resolved photoluminescence (TRPL) measurements were carried out by using time-correlated, single-photon counting technique (Hamamatsu, C10627) equipped with a femtosecond mode-locked Ti:sapphire laser (SpectraPhysics, MAITAI XF-IMW). The wavelength of excitation is 405 nm with an intensity of 1.3 mW. The space-charge limited current (SCLC) test was performed in dark condition by using a Keithley 2420 source meter (from −0.1 to 2.25 V). The electrochemical impedance spectroscopy (EIS) measurement data were acquired from the Autolab electrochemical working station with an amplitude of 5 mV at a fixed voltage of 1.0 V under one sun illumination (AM 1.5 irradiation). The frequency range is from 1 MHz to 100 Hz.

## Results and Discussion

**Fig. 1:**
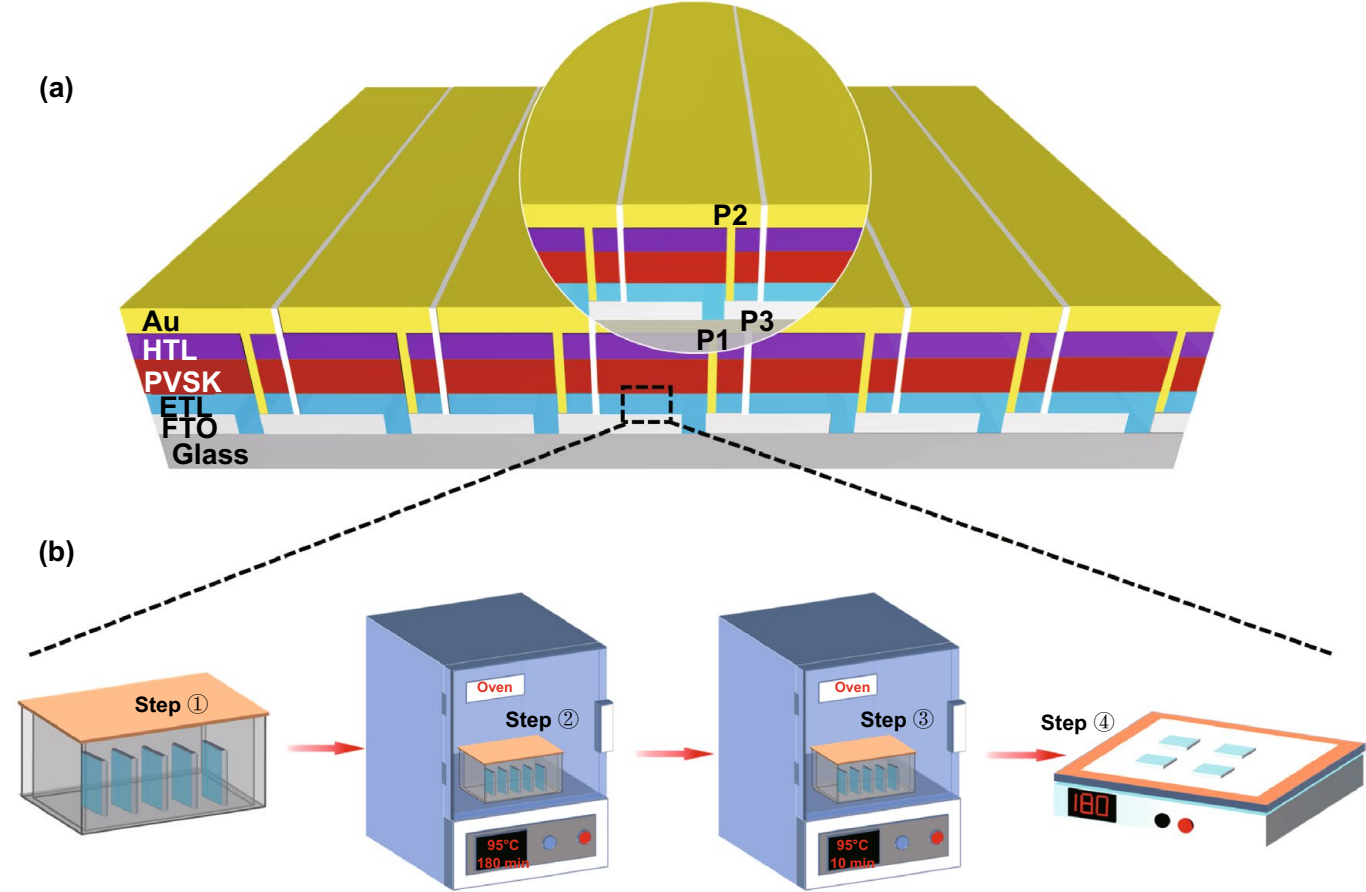
**a** Illustration of perovskite solar module architecture (ETL/HTL is electron/hole transport layer. PVSK is the perovskite layer.). **b** Schematic illustration of the SnO_2_ films fabricated by chemical bath deposition

Figure 1 illustrates the fabrication process of SnO_2_-ETL films in the case of PSM by the CBD method. For SnO_2_/K-ETL film, urea, SnCl_2_·2H_2_O, mercaptoacetic acid and hydrochloric acid (HCl) were first dissolved in deionized water as the precursor solution (Step ①). The glassware containing precursor solution and FTO substrates was heated to 95 °C and kept for 3 h in the oven (Step ②). Then, the KMnO_4_ was added into the glassware (Step ③). After 10 min continuous heating, the samples were taken out followed by post-annealing at 180 °C (1 h) on a hotplate (Step ④) (More details can be found in the experimental procedures). For SnO_2_-ETL film, the process only includes the step ①, ② and ④. It is noted that the acidic environment (**Fig. S1**) in the precursor solution makes the following well-known chemical reaction possible, when the KMnO_4_ was added into precursor solution.

1 $${\text{2KMnO}}_{{\text{4}}} {\text{ + 5SnCl}}_{{\text{2}}} {\text{ + 16HCl}} \to {\text{2KCl + 2MnCl}}_{{\text{2}}} {\text{ + 5SnCl}}_{{\text{4}}} {\text{ + 8H}}_{{\text{2}}} {\text{O}}$$

2 $${\text{SnCl}}_{4} + \left( {{\text{x}} + 2} \right){\text{ H}}_{2} {\text{O}} \to {\text{SnO}}_{2} \cdot {\text{xH}}_{2} {\text{O}} + 4{\text{HCl}}$$

The products of KCl and MnCl_2_ generated from the above chemical reaction can be deposited on the surface of SnO_2_ film in step ③ process.

**Fig. 2:**
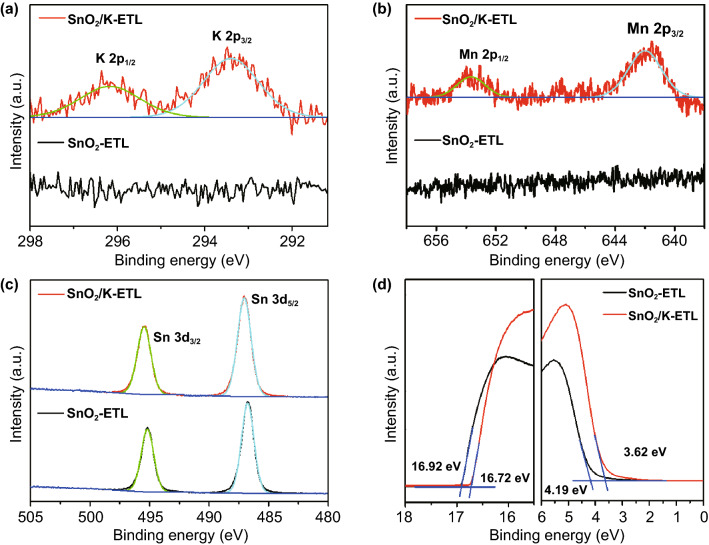
Characterization of SnO_2_ and SnO_2_/K Films. XPS spectra of the SnO_2_-ETL and SnO_2_/K-ETL films. **a** K 2p, **b** Mn 2p (*B*) and **c** Sn 3d. **d** UPS spectra acquired on the SnO_2_-ETL and SnO_2_/K﻿-ETL films

To identify our hypothesis, *X*-ray photoelectron spectroscopy (XPS) was employed to characterize the surface property changes of SnO_2_-ETLs with/without KMnO_4_ additive. The typical peaks of K 2p and Mn 2p orbitals appeared in the SnO_2_/K-ETL samples confirming that potassium and manganese are successfully deposited on the surface of SnO_2_ layer. The binding energy of 642.0 eV for Mn 2p_3/2_ is assigned to MnCl_2_ in SnO_2_/K-ETL film [45]. These results are well consistent with our hypothesis in Eq. 1. The two spin–orbit splitting peaks of Sn 3d in the SnO_2_-ETL in Fig. 2c shift from 495.1 and 486.8 eV to high binding energies of 495.5 and 487.1 eV (SnO_2_/K-ETL), which indicates more Sn (II) conversion to Sn (IV) [46, 47]. In parallel, the Cl ions are inevitably adsorbed on the top of SnO_2_ film because of the precursor solution containing SnCl_2_ and HCl. However, the increased intensity of Cl 2p in Fig. S2 suggests that large amounts of Cl ions as the products are deposited on the surface in the case of SnO_2_/K-ETL film due to the formation of KCl and MnCl_2_, which is consistent with our hypothesis in Eqs. 1 and 2.

To further evaluate the quality of SnO_2_-ETLs, the space-charge limited current (SCLC) was conducted to evaluate the carrier mobility in the case of SnO_2_ and SnO_2_/K-ETLs based on a sandwich structure of FTO/SnO_2_/Au (More details are found in experimental procedures) [39]. As seen in Fig. S3, the carrier mobility of SnO_2_ is enhanced from SnO_2_-ETL (1.62 × 10^−3^ cm^2^ V^−1^ S^−1^) to SnO_2_/K-ETL (3.91 × 10^−3^ cm^2^ V^−1^ S^−1^) after KMnO_4_ incorporation.

The electronic structures of SnO_2_-ETLs were also determined by using the ultraviolet photoemission spectroscopy (UPS) measurement as seen in Fig. 2d. The ionization energy (IE) values of samples can be extracted by the formula of IE = 21.22−E_cutoff_ + E_onset_ [44, 48]. The corresponding values of SnO_2_-ETL and SnO_2_/K-ETL films are 8.49 and 8.12 eV, respectively, which suggests the presence of potassium and manganese ions affects the energy level of SnO_2_ films. In combination with the optical bandgap of SnO_2_ films (Fig. S4), the corresponding electron affinity (EA) values of SnO_2_ films with/without KMnO_4_ are 4.12 and 4.53 eV, respectively.

**Fig. 3:**
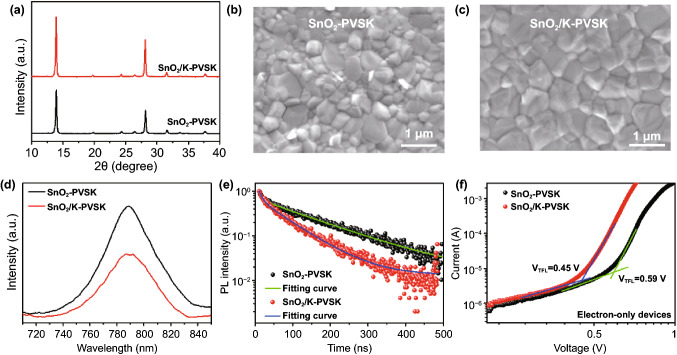
Characterization of Perovskite Films Based on SnO_2_ and SnO_2_/K Substrates. **a** *X*-ray diffraction patterns of the perovskite films deposited on SnO_2_ and SnO_2_/*K* substrates. Top view of SEM images of the perovskite films deposited on **b** SnO_2_ substrates and **c** SnO_2_/K substrates. **d** PL spectra and **e **TRPL curves of the perovskite films deposited on SnO_2_ and SnO_2_/K substrates. **f** Dark *I–V* curves of the perovskite films based on electron-only devices

Except the modification of the SnO_2_ layer, the incorporation of KMnO_4_ also has the influence on the perovskite films because the diffusion of K ions into perovskites and Mn doping has been previously demonstrated [42, 49, 50]. Previously, Zhu, Tan and coworkers demonstrated that K ions in the ETL could diffuse into the perovskite active layer to enlarge the grain size and passivate GBs [42]. Moreover, Qi et al. also discovered that partial Mn ions doping in perovskites can improve the crystallinity, morphology and phase stability of perovskites [49]. In addition to the K and Mn ions diffusing into perovskites, several previous works also demonstrated that the direct incorporation of K and Mn ions into perovskite precursors can improve the perovskite grain size, reduce the hysteresis in resultant solar cells and enhance the thermal stability [51–53]. Although there is negligible morphology difference of SnO_2_-ETLs with/without KMnO_4_ treatment as demonstrated by atomic force microscopy (AFM) images and scanning electron microscopy (SEM) images (Figs. S5 and S6). The crystallinity and surface morphology of the perovskite films grown on SnO_2_ and SnO_2_/K substrates (they are abbreviated as SnO_2_-PVSK and SnO_2_/K-PVSK, respectively) are quite different as illustrated by X-ray diffraction (XRD) and SEM (Figs. 3a-c). The compact and dense perovskite films showed high crystallinity. The enlarged grain size and the increase in XRD intensity of the perovskite film on SnO_2_/K substrate (Fig. S7) can be explained by the diffusion of K and Mn ions from the surface of the SnO_2_ layer to the bulk perovskite film in the fabrication process, which promote the growth of the perovskite grains and reduce the GBs [38, 42, 49, 50].

To further confirm it, the elemental distributions of K and Mn ions were characterized using secondary ion mass spectroscopy (SIMS) depth profiling on the FTO/SnO_2_/perovskite films (with/without annealing) based on SnO_2_/K-ETL. SIMS results in Fig. S8 evidenced K and Mn ions are located at the interface between perovskite and SnO_2_/K-ETL before annealing and diffused into in the bulk perovskite film after post-annealing. In addition, UPS and absorption spectra were conducted to identify the energy level alignments in SnO_2_-PVSK and SnO_2_/K-PVSK samples. It is found that the IE of perovskite films based on SnO_2_ and SnO_2_/K is the same (IE = 5.65 eV). In combination with the bandgap of the perovskite films, the EA value is 4.10 eV (Figs. S9 and S10). It is noted that the reduced electron barrier between perovskite and SnO_2_/K-ETL layer in comparison with perovskite/SnO_2_-ETL interface can accelerate the carrier extraction (Fig. S10). Figures 3d-e show the steady-state photoluminescence (PL) and time-resolved PL (TRPL) of perovskites on FTO/SnO_2_ substrates with/without KMnO_4_ treatment. The high fluorescence quenching efficiency of SnO_2_/K-PVSK indicates the efficient electron extraction occurred from perovskite to SnO_2_/K-ETL layer because of the high carrier mobility of SnO_2_/K in comparison with SnO_2_ [12]. The lifetimes of the perovskite films were fitted by a bi-exponential function of $\tau _{{{\text{average}}}} = \frac{{A_{1} \tau _{1}^{2} + A_{2} \tau _{2}^{2} }}{{A_{1} \tau _{1} + A_{2} \tau _{2} }}$ [44, 54]. In this equation, *A*_i_ (i = 1, 2) is the amplitude. The τ_1_ of the fast decay component represents the nonradiative recombination. Similarly, τ_2_ represents the radiative recombination with a slow decay component. Compared with SnO_2_-PVSK sample, the short lifetime of the SnO_2_/K-PVSK (Table S2) indicates the less defects and efficient carrier extraction because of the reduced electron barrier at the perovskite/SnO_2_ interface (Fig. S10), and the high carrier mobility of SnO_2_ in the case of SnO_2_/K-ETL [12]. To further investigate the influence of KMnO_4_ on the perovskites and perovskite/SnO_2_ interface, the dark *I*-*V* measurement was conducted by assembling an electron-only devices with a configuration of FTO/SnO_2_/perovskite/PCBM/C_60_/Au. The SCLC equation was used to calculate the trap density of the perovskite films as follows [20, 44, 55]:

3 $$V_{{{\text{TFL}}}} = \frac{{{\text{en}}_{{{\text{trap}}}} L^{2} }}{{2\varepsilon \varepsilon _{0} }}$$

where *V*_TFL_ represents the onset voltage of the trap filled limit. $\varepsilon _{0}$ and $\varepsilon$ are the constant of vacuum permittivity in free space and the relative dielectric constant of perovskite. e is electric charge. *L* is the thickness of the perovskite film (Fig. S11). The reduced trap defects of SnO_2_/K-PVSK (2.94 × 10^15^ cm^−3^) compared with SnO_2_-PVSK (3.76 × 10^15^ cm^−3^) suggest the presence of K and Mn ions can effectively improve the crystallinity of perovskite film and reduce the defects.

**Fig. 4:**
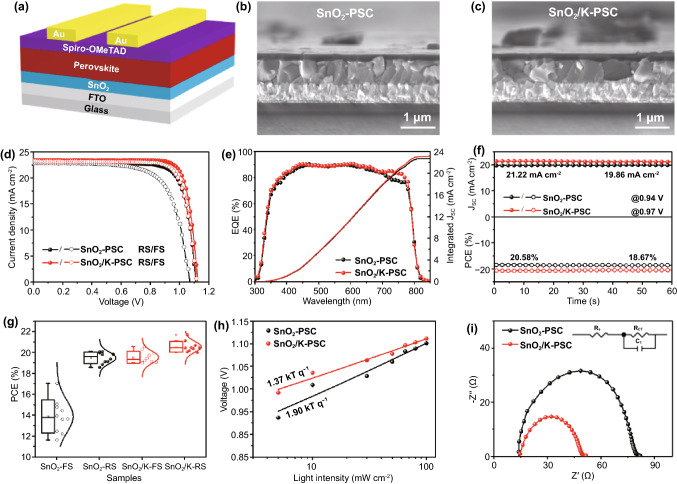
Characterization and Performance of Lab-Scale Perovskite Solar Cells. **a** Schematic drawing showing the perovskite solar cell device structure. Cross section SEM images of the PSCs based on **b** SnO_2_-ETLs and **c** SnO_2_/K-ETLs. **d** *J-V* curves, **e** EQE spectra and **f** the steady-state power output performance of PSCs based on SnO_2_ and SnO_2_/K-ETLs. **g** Statistical distribution of the solar cell performances. **h** The dependence of open-circuit voltage on light intensity for the PSCs based on SnO_2_ and SnO_2_/K-ETLs. **i** Nyquist plots of the PSCs based on SnO_2_ and SnO_2_/K-ETLs. The inset shows the equivalent circuit diagram

Figure 4a illustrates the structure of planar PSCs based on SnO_2_ and SnO_2_/K (they are abbreviated as SnO_2_-PSC and SnO_2_/K-PSC, respectively). Typical cross section SEM images of devices in Figs. 4b, c and S12 show uniform and compact perovskite layers. A relatively low PCEs of 20.09% and 17.05% for SnO_2_-PSC under reverse scan (RS) and forward scan (FS) exhibited a large hysteresis. As the amount of KMnO_4_ is increased, the photovoltaic performance of devices increases as shown in Fig. S13 and Table S3. The champion PCE is achieved at a concentration of 8 mM, beyond which the performance starts to decrease. Moreover, the hysteresis (hysteresis index (HI) = PCE_RS_/PCE_FS_) [56] is also improved with the introduction of KMnO_4_. The HI decreases from 1.18 for 0 mM KMnO_4_ to 1.05 for 8 mM KMnO_4_. Figure 4d shows the photovoltaic performance of the best-performing SnO_2_-PSCs and SnO_2_/K-PSCs. Compared to SnO_2_ based device that shows a large hysteresis, the reduced hysteresis after KMnO_4_ treatment is observed with the PCE_RS_ of 21.70% and PCE_FS_ of 20.58%. The corresponding integrated J_sc_ from EQE are 22.69 and 23.11 mA cm^−2^, respectively (Fig. 4e), which are in agreement with J_sc_ extracted from *J − V* curves, with a discrepancy below 2%. It is worth noting that the enhanced EQE in the wavelength region of 700 ~ 800 nm in the case of the SnO_2_/K-based PSC is ascribed to the reduced trap density at the surface of the perovskite film and faster carrier extraction at the perovskite/HTL interface by the incorporation of K and Mn ions into the perovskites [44]. Additionally, both SnO_2_ and SnO_2_/K-based PSCs exhibit stable output (under initial maximum power point (MPP) voltage) with PCE of 18.67% and 20.58%, respectively (Fig. 4f).

Statistical analyses of the photovoltaic parameters reveal that the performance is reproducible with minimal variations and low HI of 1.05 for SnO_2_/K-PSC compared to SnO_2_ based devices with large hysteresis (HI = 1.40). (Figs. 4g, S14 and Table S4). It is well known that hysteresis is mainly originated from: [52, 57] 1) the ion migration in perovskites; 2) charge trapping/de-trapping in deep trap sites originated from defects; 3) ferroelectric polarization of perovskites. The same composition of perovskite used in the PSCs suggests that the main influence comes from issues 1) and 2). Large amounts of defects at ETL/perovskite interface serve as the recombination centers in the devices, which highly increase the nonradiative recombination, leading to a large hysteresis [57]. In parallel, the iodide ions migrating through GBs are accumulated at electrode and interface of ETL/perovskite, which increases the capacitance in the devices, resulting in a large hysteresis simultaneously [57, 58]. However, the reduced interfacial trap-assisted recombination at ETL/perovskite by decorating the interface with the KMnO_4_ treatment and the inhibition of the ion migration by potassium doping can effectively eliminate the hysteresis [57, 59]. Our results are consistent with previous findings that the presence of potassium halide layer could reduce the ionic migration, leading to a reduced hysteresis [50, 57]. In addition, the enlarged perovskite grain size and enhanced crystallinity of the perovskite film in the case of SnO_2_/K sample can also reduce the hysteresis because of less GBs and defects [60, 61].

Besides the hysteresis, we also evaluated the light-intensity-dependent V_oc_ of PSCs under increasing illumination intensities, which reflects the trap-assisted recombination [39]. As seen in Fig. 4h, the SnO_2_/K-PSC with a small slope value (1.37 kT q^−1^) compared to the SnO_2_ based device (1.90 kT q^−1^) indicates less trap-assisted recombination, which agrees well with TRPL results. Furthermore, the electrochemical impedance spectroscopy (EIS) measurements were performed under AM 1.5 G light illumination at a bias voltage of 1.0 V. By fitting the equivalent circuit diagram as seen in Fig. 4i, the closer sheet resistance (*R*_s_) of two devices suggests the same configuration in devices (Table S5). In contrast, the smaller semicircle defined as charge transport resistance (*R*_ct_) of SnO_2_/K-PSC (22.44 Ω) compared with SnO_2_-PSC (62.57 Ω) reveals the fastest carrier transport, which is ascribed to less trap density and well energy level alignment at the interface of SnO_2_/perovskite. In addition to the efficiency, the stability of devices is also an important part to evaluate the performance of devices. We first check the phase stability of perovskite that was stored in ambient air in a dry room with a relative humidity of ~ 20% without any encapsulation for 5 months. For both devices, the high intensity of the PbI_2_ peak at 12.6° is observed after storage for 5 months, which indicates that part of perovskite active materials has decomposed to PbI_2_ (Fig. S15). But when compared to the SnO_2_-PSC, the SnO_2_/K-PSC shows a substantially stronger perovskite peak at ~ 14°, suggesting that degradation of perovskite in SnO_2_/K-PSC is slower than that in SnO_2_-PSC. In addition, we also evaluated the storage stability of the resultant solar cell devices. The average efficiency of SnO_2_/K-PSC is 18.8%, which is much higher than SnO_2_-PSC with a PCE of 17.4% (Fig. S16 and Table S6). On the basis of the above analyses, we conclude that SnO_2_/K-ETL can improve the phase stability of perovskite.

**Fig. 5:**
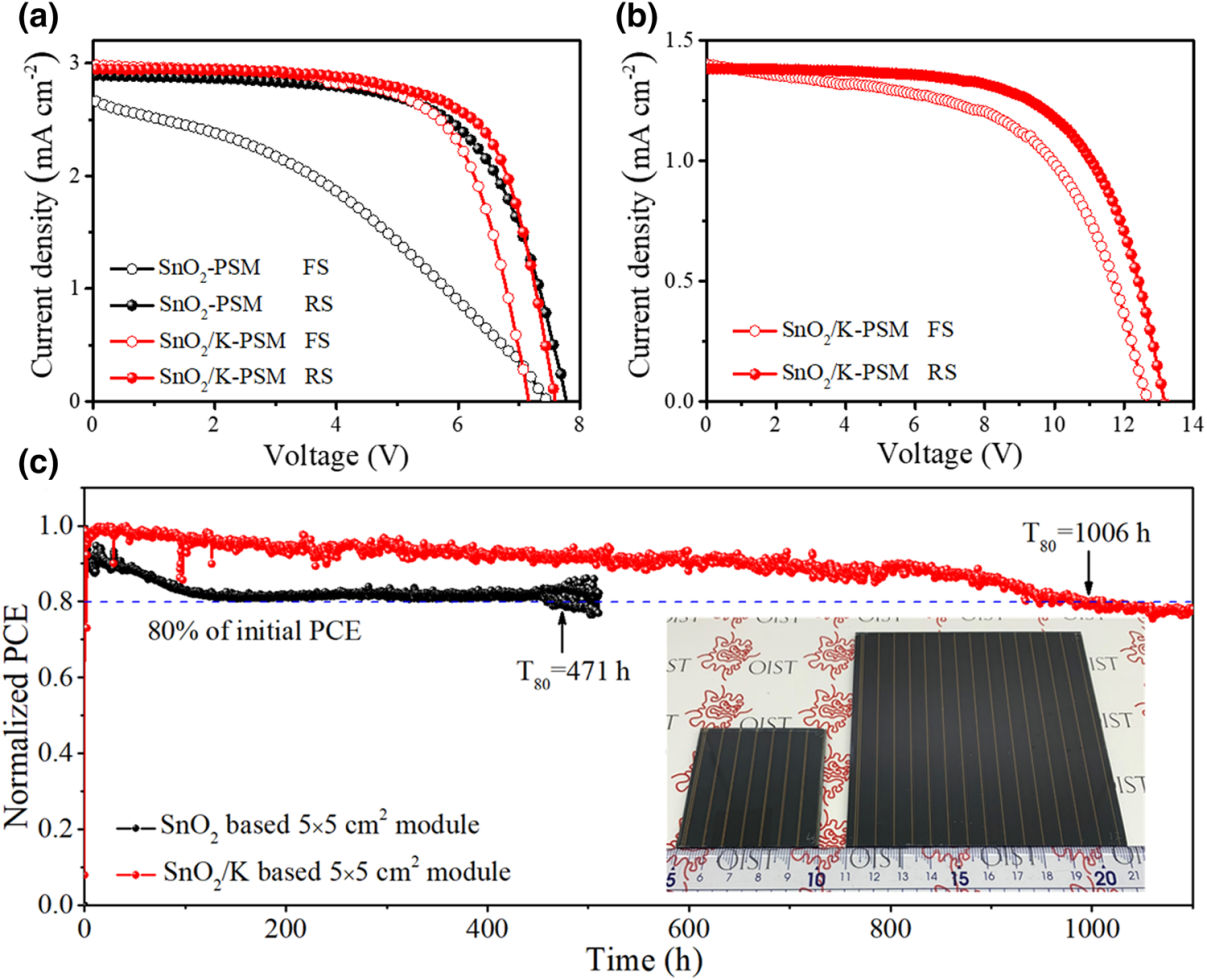
Performance of Perovskite Solar Cell Modules. **a** *J-V* curves of 5 × 5 cm^2^ PSMs based on SnO_2_ with/without KMnO_4_ passivation under forward and reverse scan. b J-V curves of 10 × 10 cm^2^ PSMs based on SnO_2_/K under forward and reverse scan. **c** Operational stability of 5 × 5 cm^2^ PSMs based on SnO_2_/K with encapsulation under a steady applied voltage and constant illumination (AM 1.5G, 100 mW cm^−2^), inset: photographs of 5 × 5 cm^2^ and 10 × 10 cm^2^ PSMs

To highlight the benefits of this approach for fabricating SnO_2_ layer, we also demonstrated its feasibility to be up-scalable (Figs. 5c (inset) and S17). Statistical analyses of photovoltaics performance based on 5 × 5 cm^2^ PSMs (10 devices) with a designed area of 22.4 cm^2^ (Fig. S18a) showed an average PCE of 11.97 ± 1.16% and 13.62 ± 0.87% for SnO_2_-PSMs and SnO_2_/K-PSMs, respectively (Fig. S19, Tables S7-S8). The champion module PCEs of 15.62% with a *V*_oc_ of 7.59 V, a *J*_sc_ of 2.95 mA cm^−2^ and a FF of 0.699 under reverse scan and 14.25% with a *V*_oc_ of 7.17 V, a *J*_sc_ of 2.98 mA cm^−2^ and a FF of 0.697 under forward scan for SnO_2_/K-PSM show a reduced hysteresis (HI = 1.10) compared with SnO_2_-PSM showing a large hysteresis (HI = 1.95) with module PCEs of 14.58% and 7.49% under RS and FS, respectively (Fig. 5a, Table S9). It is worth to note that the active area PCE for the champion devices is up to 17.26% with a geometric fill factor (GFF) = 90.5% (Fig. S18b), which is higher than previous related reports (Table S10). Furthermore, 10 × 10 cm^2^ PSMs with a designed area of 91.8 cm^2^ by employing SnO_2_/K as ETL were fabricated with an average PCE of 10.06 ± 0.99% (Fig. S20 and Table S11). The best module generates a PCE of 11.80% (*V*_oc_ of 13.16 V, *J*_sc_ of 1.38 mA cm^−2^ and *FF* of 0.648) under reverse scan and 10.17% (*V*_oc_ of 12.65 V, *J*_sc_ of 1.40 mA cm^−2^ and *FF* of 0.574) under forward scan (Fig. 5b). The corresponding active area PCE is over 13.72% with a GFF of 86.0% (Fig. S21).

In addition, the operation stability of 5 × 5 cm^2^ PSM (with UV-curing polymer encapsulation) in Figs. 5c and S22 was evaluated under continuous AM 1.5G light illumination at a fixed bias that is determined by the initial maximum power point from *J-V* curves in ambient condition (RH ~ 55%, 25 °C). A fast decay in the performance of SnO_2_-PSM is observed, resulting in a poor operational stability with a T_80_ lifetime (the lifespan of the solar module PCE drops to 80% of its initial performance) of 471 h. In contrast, the SnO_2_/K-PSM exhibits an excellent operational performance with a *T*_80_ lifetime of 1006 h. This outstanding stability is ascribed to the better contact of SnO_2_/perovskite interface and high crystallinity of the perovskite layer. Two decay processes, including a fast exponential decay regime followed by a subsequent slower linear one, are often discussed as commonly observed profiles in operational stability of PSCs [56, 62]. The exponential decay corresponds to a fast burn-in process associated with migration of cation vacancies, which induces the formation of an additional Debye layer at the interface between ETL and perovskite, inhibiting carrier extraction [62, 63]. The reduced burn-in region in the exponential decay profile in the case of SnO_2_/K-PSM indicates the ETL/perovskite interface is well passivated by KMnO_4_ treatment, reducing the accumulation of ionic defects and vacancies at the interfaces. However, severe iodide ions migration in the SnO_2_-PSM can accelerate the degradation of perovskite and the formation of recombination centers, which results in a high recombination and efficiency loss, leading to a fast decay and a poor stability [57, 58, 64]. Moreover, Mn doping also improves the stability of perovskite in the case of SnO_2_/K-PSMs in the permanent degradation regime (linear decay). The volumetric ratio between BX_6_ (*B* = Pb^2+^, *X* = I, Br) octahedra and cations will determine the phase stability of perovskites [49, 65]. The presence of Mn^2+^ ion has a smaller radius of 0.97 Å in comparison with Pb^2+^ ion (1.33 Å), which substantially induces the contraction of the BX_6_ octahedral volume, leading to a better holding of the BX_6_ octahedra by the mixed cation in the perovskite structure[49]. This volume contraction would possibly improve the stability of mixed cation, which enhances the operational stability of SnO_2_/K-PSM.

## Conclusion

In summary, we developed a facile CBD method to prepare the SnO_2_ layer in large area by introducing KMnO_4_ as an additive. High oxidant property of KMnO_4_ not only promotes the formation of Sn (IV), but also provides additional potassium and manganese ions. The presence of potassium can effectively reduce the hysteresis and enlarge perovskite grains. Meanwhile, manganese doping not only improves the crystallinity of perovskite grains, but also enhances the phase stability of perovskites. A champion PCE of 21.70% with a reduced hysteresis was obtained for lab-scale devices by this multifunctional interface engineering strategy. Importantly, this precursor solution passivation in CBD method was demonstrated as a compatible technique for upscalable fabrication of solar modules. The best module efficiency of 15.62% (active area PCE = 17.26%) and 11.80% (active area PCE = 13.72%) were achieved for 5 × 5 and 10 × 10 cm^2^ PSMs, respectively. The continuous operation stability of the SnO_2_/K-based PSM showed a T_80_ operation lifetime exceeding 1000 h in ambient condition (relative humidity 55%, 25 °C) for the encapsulated 5 × 5 cm^2^ SnO_2_/K-based PSM.

## Supplementary Information

Below is the link to the electronic supplementary material.

Supplementary file1 (PDF 1253 KB)
