# Supplementary material for: Up-Scalable Fabrication of SnO2 with Multifunctional Interface for High Performance Perovskite Solar Modules
Source: Nanomicro Lett. 2021 Jul 10;13:155. doi: 10.1007/s40820-021-00675-7 (PMC8271052; doi:10.1007/s40820-021-00675-7)
Supplement: Supplementary file 1 — Supplementary file1 (PDF 1253 KB) [file 40820_2021_675_MOESM1_ESM.pdf]

Supporting information

Up-Scalable Fabrication of SnO_2 with Multifunctional Interface for High Performance Perovskite Solar Modules

Guoqing Tong, Luis K. Ono, Yuqiang Liu, Hui Zhang, Tongle Bu, and Yabing Qi*

Energy Materials and Surface Sciences Unit (EMSSU)

Okinawa Institute of Science and Technology Graduate University (OIST)

1919-1 Tancha, Onna-son, Kunigami-gun, Okinawa 904-0495, Japan

*Corresponding author: Yabing Qi, E-mail: Yabing.Qi@OIST.jp

List of Contents

1. Supplementary Figures

Fig. S1 pH value of the CBD-SnO_2 precursor solution after heating at 95 °C for 3 hours (as depicted in Fig. 1b, Step ②).

Fig. S2 XPS spectra of the Cl 2p core-level region on SnO_2-ETL and SnO_2/K-ETL films.

Fig. S3 Dark I–V curves of the SnO_2 films based on the FTO/SnO_2/Au structure.

Fig. S4 The relationship of $(\alpha h\nu)^2$ vs energy ($h\nu$) for the SnO_2-ETL film and SnO_2/K-ETL film.

Fig. S5 SEM images of FTO, SnO_2-ETL and SnO_2/K-ETL films.

Fig. S6 Tapping-mode AFM images of (a) FTO substrate, (a) SnO_2-ETL film and (b) SnO_2/K-ETL film.

Fig. S7 Grain size distributions of the perovskite films on (a, c) the SnO_2 and (b, d) SnO_2/K substrates. Scale bars are all 1 μm.

Fig. S8 The distribution of K, Mn, Sn and Pb ions determined from the secondary ion mass spectrometry (SIMS) measurement for the perovskite film (a) without and with annealing.

Fig. S9 (a) Absorbance of the perovskite films based on the SnO_2 and SnO_2/K substrates. (b) The corresponding optical bandgap of the perovskite films deduced by the Kubelka-

Munk equations. UPS spectra of (c) the valence features, and (d) secondary electron onset of the perovskite films based on the SnO_2 and SnO_2/K substrates.

Fig. S10 The energy level diagram for SnO_2-ETL, SnO_2/K-ETL and perovskite.

Fig. S11 Thickness of the perovskite film determined by the cross-section SEM image.

Fig. S12 Low magnification cross-section SEM images of the PSCs based on the SnO_2 with/without $KMnO_4$ treatment.

Fig. S13 J-V curves of the SnO_2 based PSCs with different amounts of $KMnO_4$.

Fig. S14 Statistical distribution of SnO_2-PSCs and SnO_2/K-PSCs based on 10 devices. (a) V_{oc}, (b) J_{sc} and (c) FF.

Fig. S15 XRD of SnO_2-PSC and SnO_2/K-PSCs after storage for 5 months in ambient air in a dry room with a relative humidity of ~20% without encapsulation.

Fig. S16 J-V curves of the SnO_2-PSCs and SnO_2/K-PSCs based on 10 devices after 5 months storage in ambient air in a dry room with a relative humidity of ~20% without encapsulation.

Fig. S17 Schematic drawing showing the side view of the perovskite solar module showing the interconnections including P1, P2, P3 lines.

Fig. S18 (a) Top view of the 5 × 5 cm^2 FTO substrate pattern. (b) Optical photograph of the sub-cell separation including P1, P2 and P3 patterns in the 5 × 5 cm^2 SnO_2/K-PSM. The corresponding geometric fill factor (GFF) is determined to be approximately 0.905.

Fig. S19 J-V curves of the 5 × 5 cm^2 PSMs based on 10 devices under reverse scan for (a) SnO_2-PSMs. (b) SnO_2/K-PSMs.

Fig. S20 J-V curves of the 10 × 10 cm^2 SnO_2/K-PSMs based on 10 devices under reverse scan.

Fig. S21 (a) Top view of the 10 × 10 cm^2 FTO substrate pattern. (b) Optical photograph of the sub-cell separation including P1, P2 and P3 patterns in 10 × 10 cm^2 SnO_2/K-PSM. The corresponding GFF is determined to be approximately 0.860.

Fig. S22 Operational stability of the 5 × 5 cm^2 PSMs with encapsulation under a steady applied voltage and constant illumination (AM 1.5G, 100 mW cm^{-2}).

2. Supplementary Tables

Table S1 The comparison of the efficiency and active area of perovskite solar cells by employing chemical bath deposition.

Table S2 Fast and slow components for the TRPL decay.

Table S3 Photovoltaics parameters of the SnO_2 based PSCs with different amounts of $KMnO_4$.

Table S4 Statistical photovoltaic parameters of open-circuit voltage (V_{oc}), short-circuit photocurrent density (J_{sc}), fill factor (FF) and power conversion efficiency (PCE) of the SnO_2-PSCs and SnO_2/K-PSCs based on 10 devices.

Table S5 Fitting parameters of the EIS measurement of the PSCs based on the SnO_2 and SnO_2/K substrates.

Table S6 Statistical photovoltaic parameters of open-circuit voltage (V_{oc}), short-circuit photocurrent density (J_{sc}), fill factor (FF) and power conversion efficiency (PCE) of the SnO_2-PSCs and SnO_2/K-PSCs based on 10 devices after 5 months storage in ambient air in a dry room with a relative humidity of ~20% without any encapsulation.

Table S7 Photovoltaics parameters of 10 SnO_2 based perovskite solar modules (5 × 5 cm^2 PSMs) under reverse scan.

Table S8 Photovoltaics parameters of 10 SnO_2/K based perovskite solar modules (5 × 5 cm^2 PSMs) under reverse scan.

Table S9 Photovoltaics parameters of the champion efficiency of the SnO_2/K based perovskite solar modules (5 × 5 cm^2 PSMs).

Table S10 The comparison of the efficiency and operational stability of perovskite solar modules with the area over 20 cm^2.

Table S11 Photovoltaics parameters of 10 SnO_2/K based perovskite solar modules (10 × 10 cm^2 PSMs) under reverse scan.

Figures

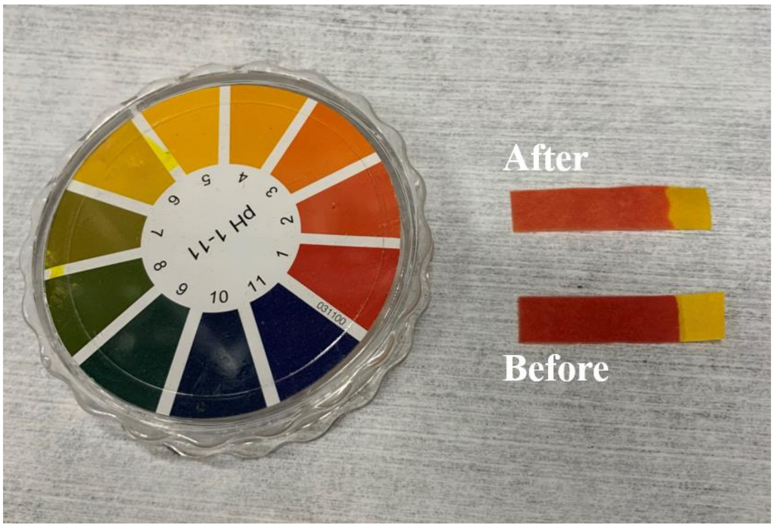

Fig. S1 pH value of the CBD-SnO_2 precursor solution after heating at 95 °C for 3 hours (as depicted in Fig. 1b, Step ②)

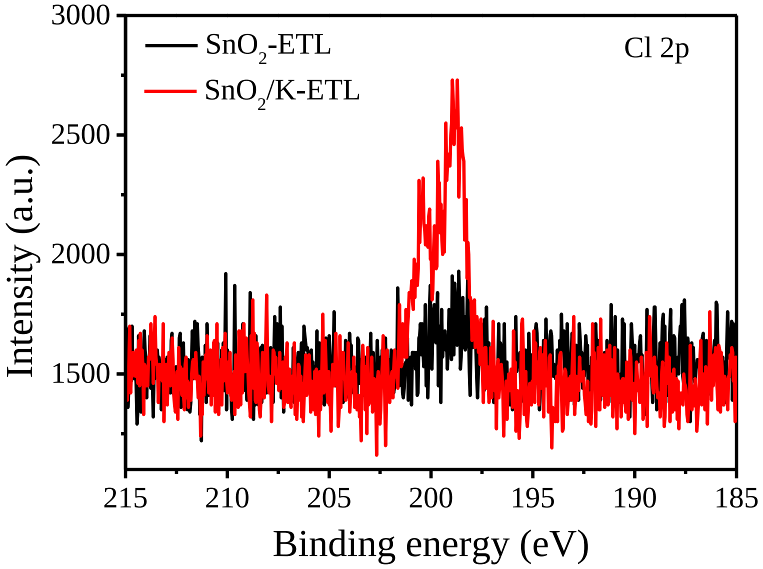

Fig. S2 XPS spectra of the Cl 2p core-level region on SnO_2-ETL and SnO_2/K-ETL films.

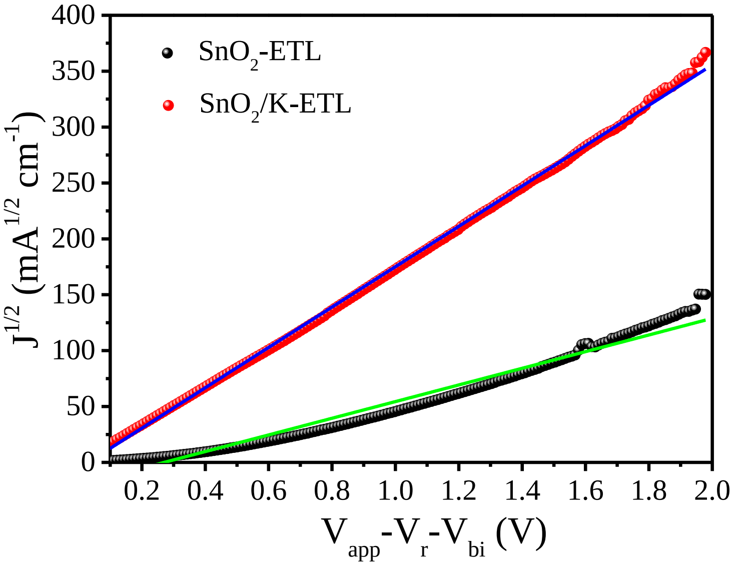

Fig. S3 Dark I–V curves of the SnO_2 films based on the FTO/SnO_2/Au structure.

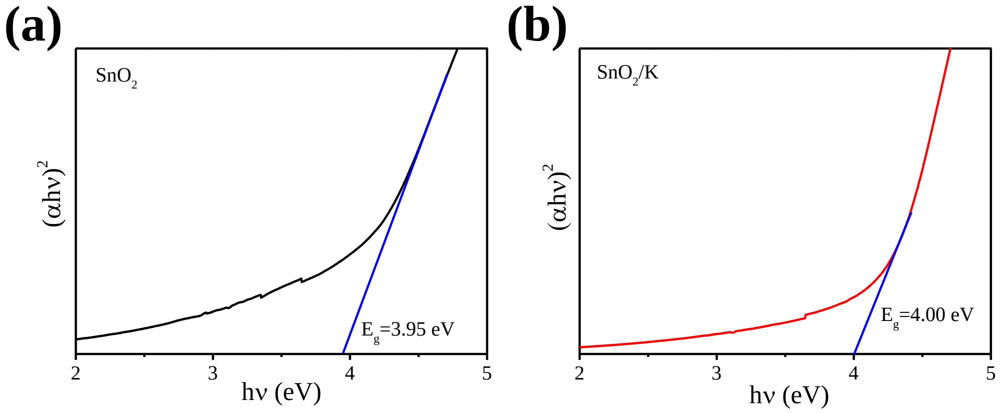

Fig. S4 The relationship of $(\alpha h\nu)^2$ vs energy ($h\nu$) for the SnO_2-ETL film and SnO_2/K-ETL film.

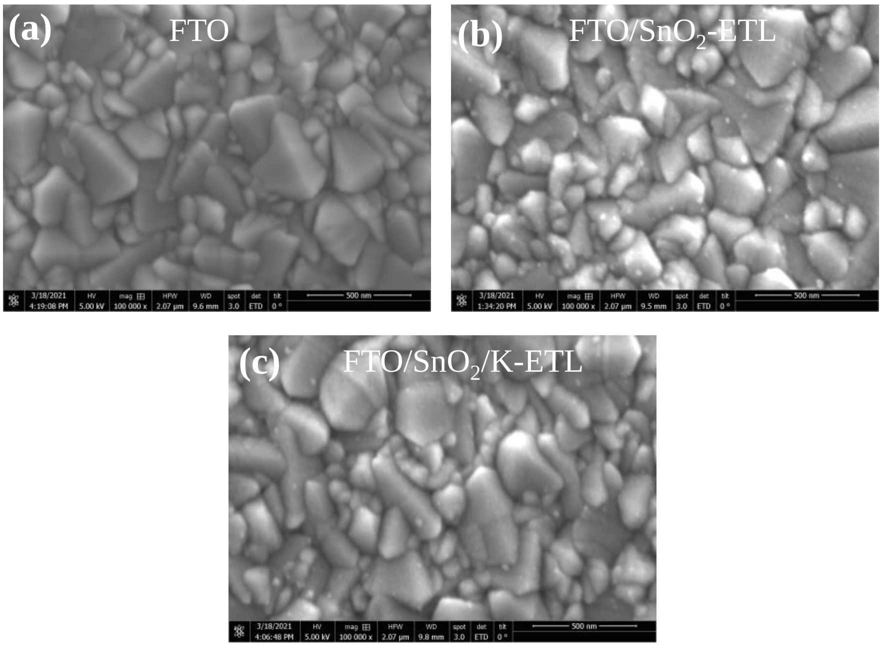

Fig. S5 SEM images of FTO, SnO_2-ETL and SnO_2/K-ETL films.

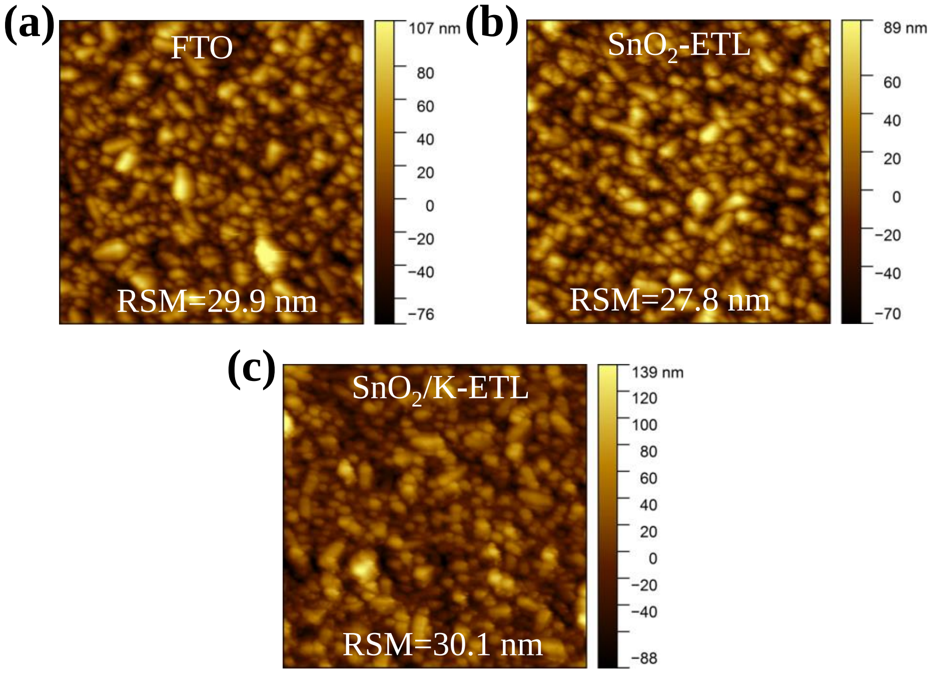

Fig. S6 Tapping-mode AFM images of (a) FTO substrate, (a) SnO_2-ETL film and (b) SnO_2/K-ETL film.

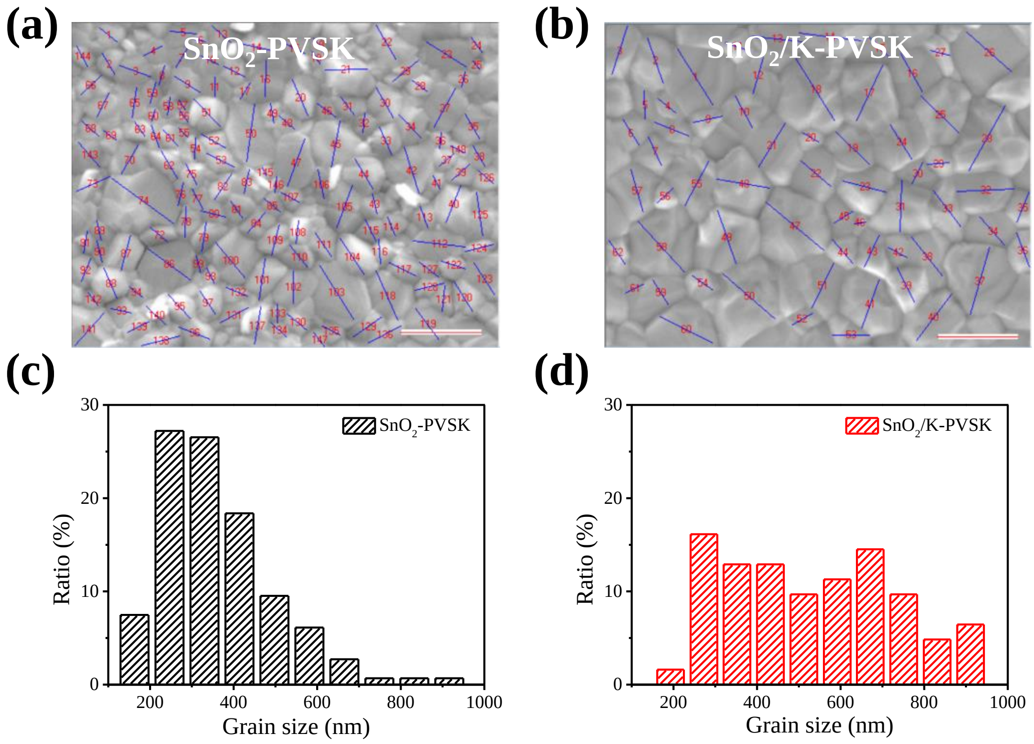

Fig. S7 Grain size distributions of the perovskite films on (a, c) the SnO_2 and (b, d) SnO_2/K substrates. Scale bars are all 1 μm.

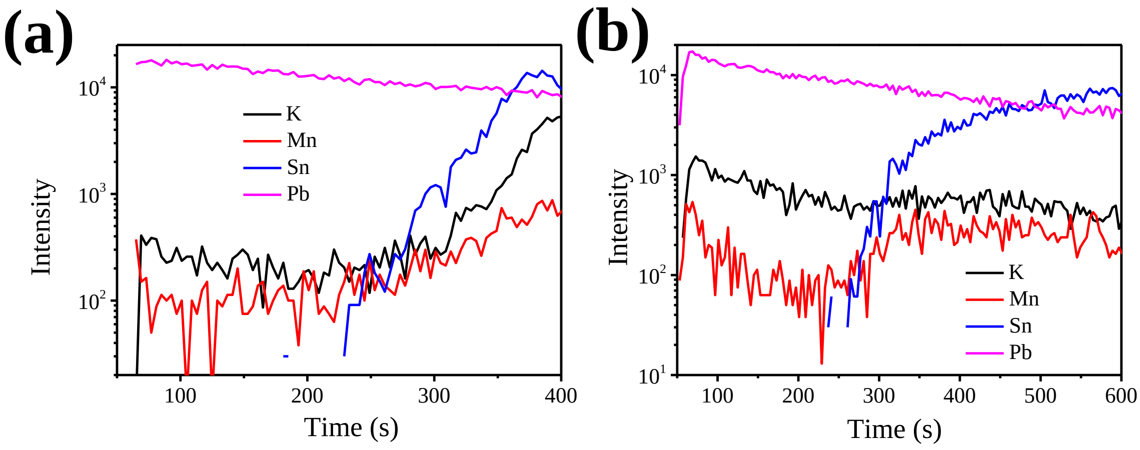

Fig. S8 The distribution of K, Mn, Sn and Pb ions determined from the secondary ion mass spectrometry (SIMS) measurement for the perovskite film (a) without and with annealing.

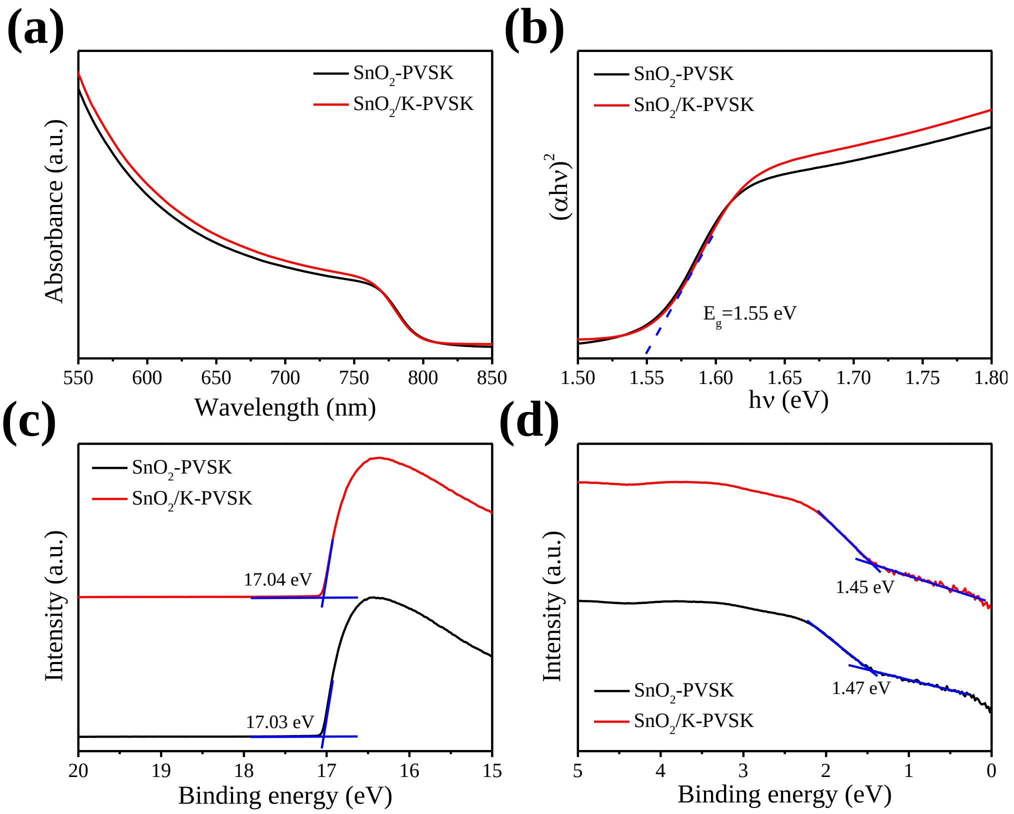

Fig. S9 (a) Absorbance of the perovskite films based on the SnO_2 and SnO_2/K substrates. (b) The corresponding optical bandgap of the perovskite films deduced by the Kubelka-Munk equations. UPS spectra of (c) the valence features, and (d) secondary electron onset of the perovskite films based on the SnO_2 and SnO_2/K substrates.

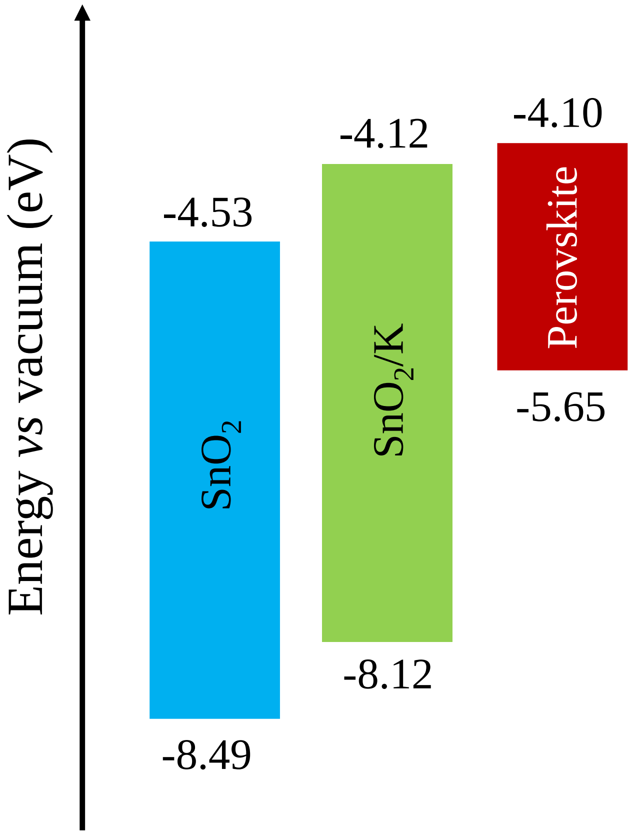

Fig. S10 The energy level diagram for SnO_2-ETL, SnO_2/K-ETL and perovskite.

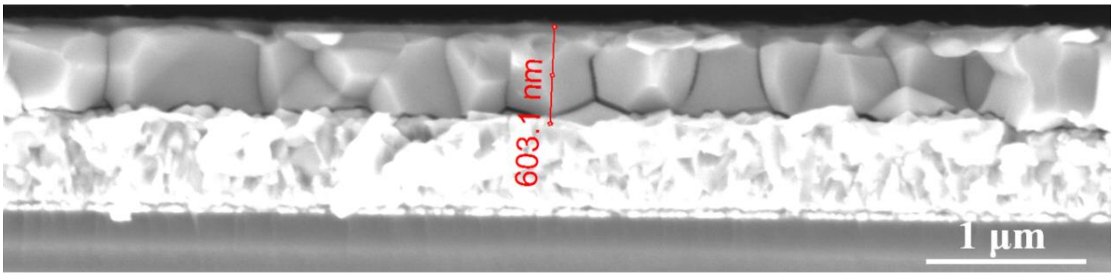

Fig. S11 Thickness of the perovskite film determined by the cross-section SEM image.

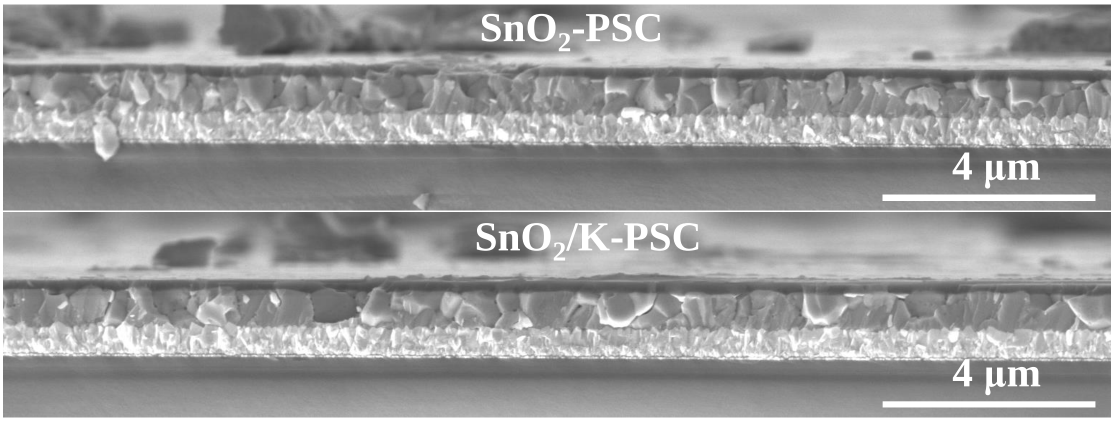

Fig. S12 Low magnification cross-section SEM images of the PSCs based on the SnO_2 with/without $KMnO_4$ treatment.

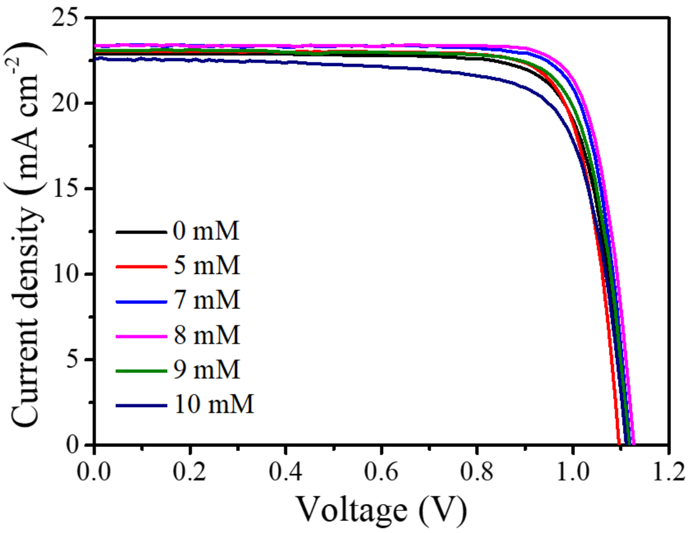

Fig. S13 J-V curves of the SnO_2 based PSCs with different amounts of $KMnO_4$.

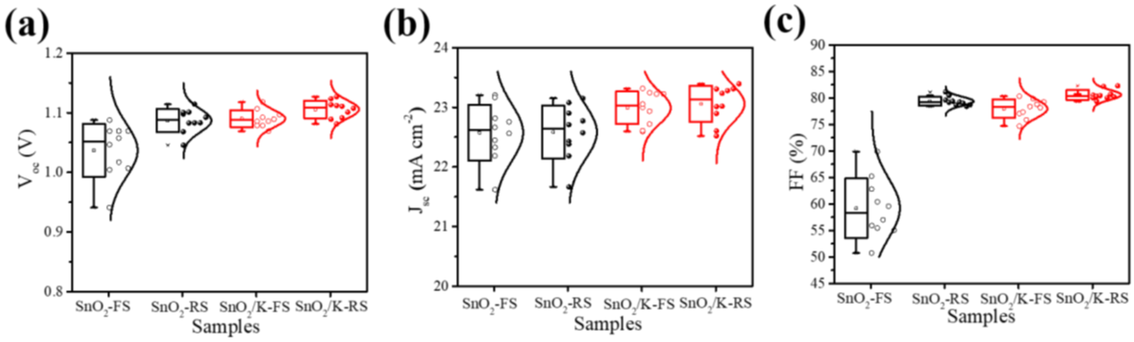

Fig. S14 Statistical distribution of SnO_2-PSCs and SnO_2/K-PSCs based on 10 devices. (a) V_{oc}, (b) J_{sc} and (c) FF.

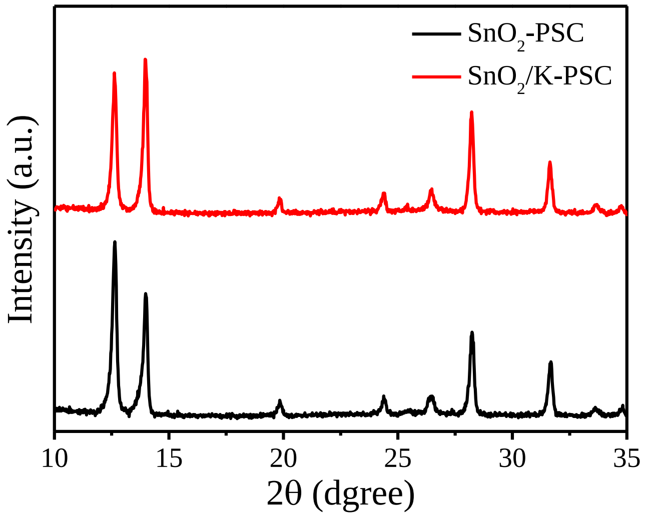

Fig. S15 XRD of SnO_2-PSC and SnO_2/K-PSCs after storage for 5 months in ambient air in a dry room with a relative humidity of ~20% without encapsulation.

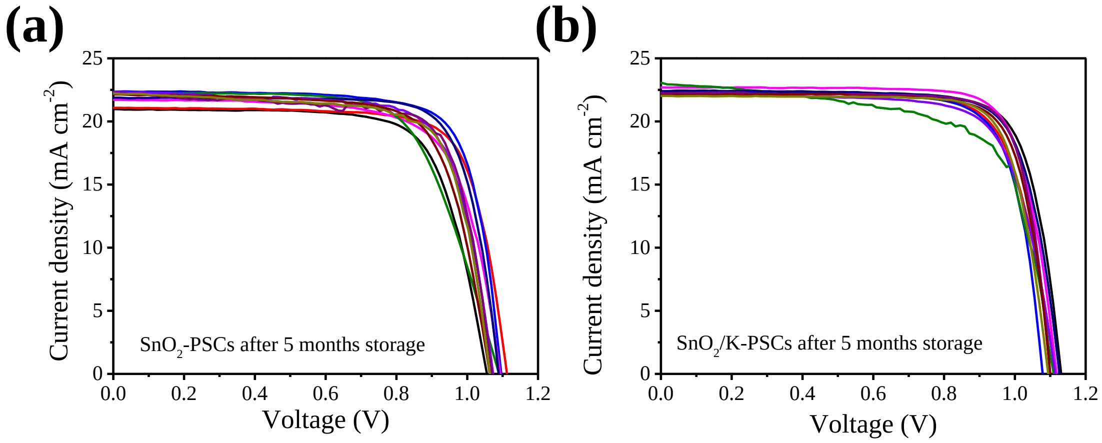

Fig. S16 J-V curves of the SnO_2-PSCs and SnO_2/K-PSCs based on 10 devices after 5 months storage in ambient air in a dry room with a relative humidity of ~20% without encapsulation.

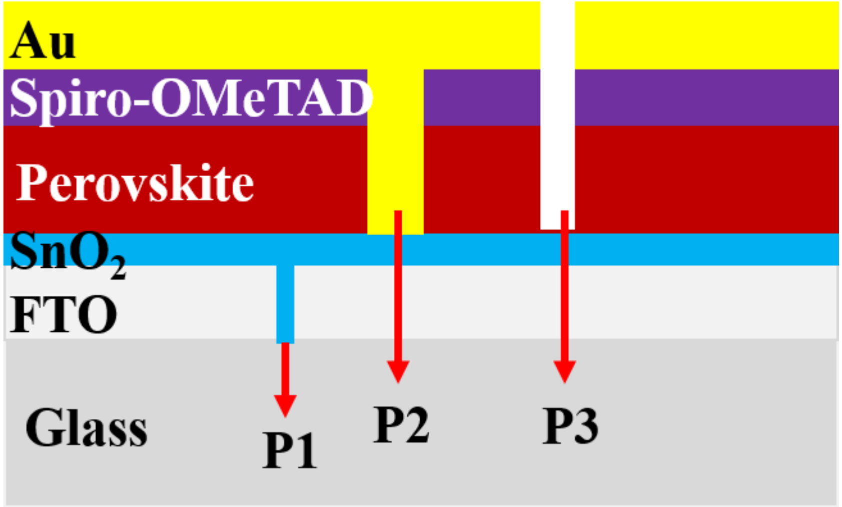

Fig. S17 Schematic drawing showing the side view of the perovskite solar module showing the interconnections including P1, P2, P3 lines.

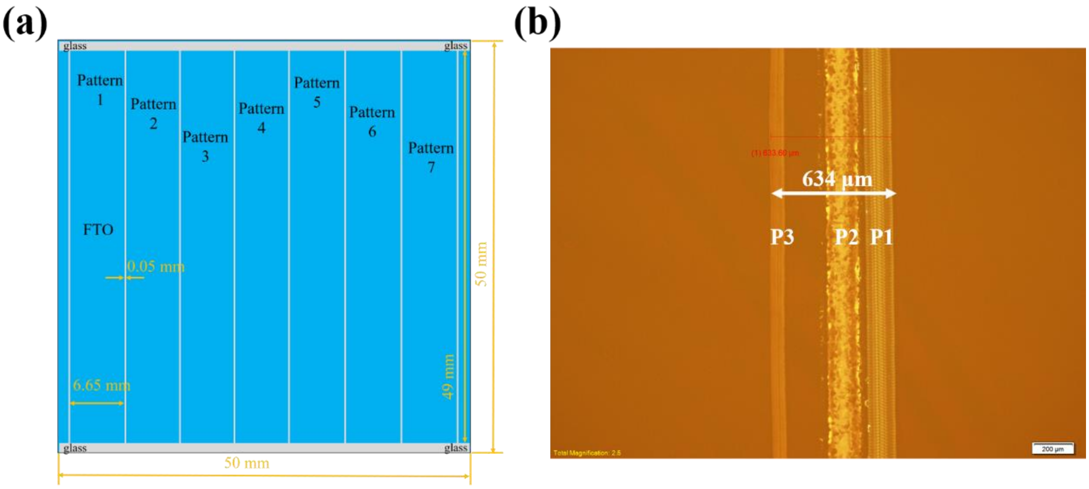

Fig. S18 (a) Top view of the 5 × 5 cm^2 FTO substrate pattern. (b) Optical photograph of the sub-cell separation including P1, P2 and P3 patterns in the 5 × 5 cm^2 SnO_2/K-PSM. The corresponding geometric fill factor (GFF) is determined to be approximately 0.905.

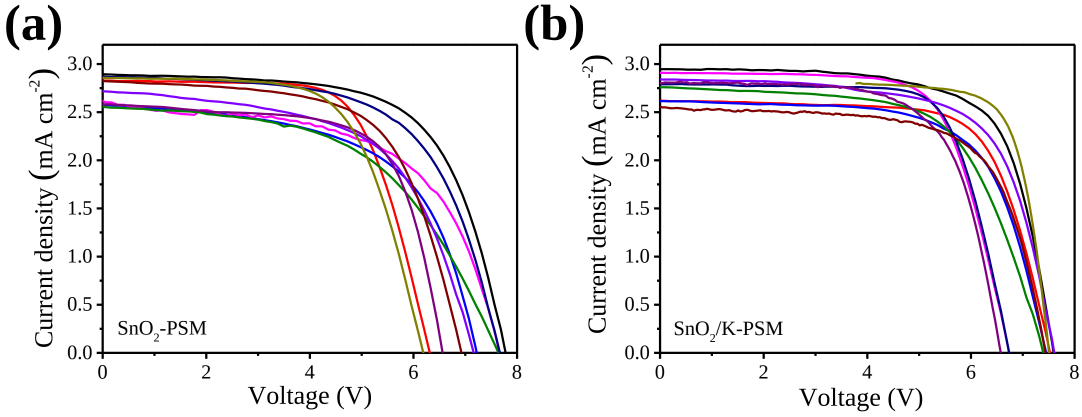

Fig. S19 J-V curves of the 5 × 5 cm^2 PSMs based on 10 devices under reverse scan for (a) SnO_2-PSMs. (b) SnO_2/K-PSMs.

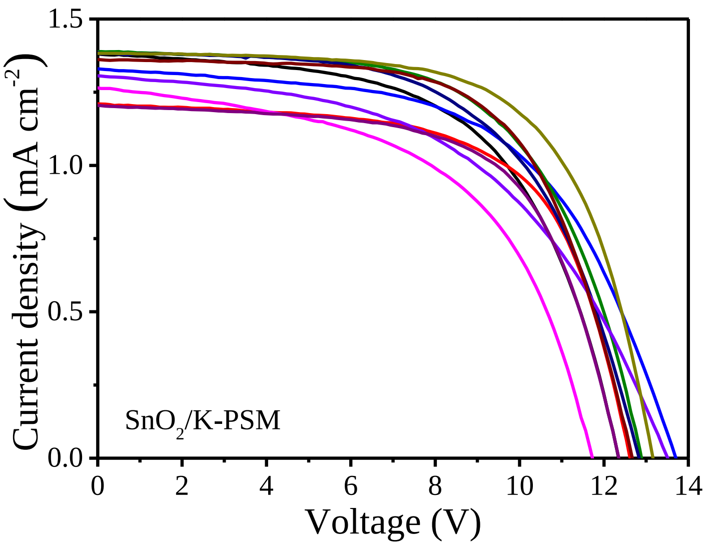

Fig. S20 J-V curves of the 10 × 10 cm^2 SnO_2/K-PSMs based on 10 devices under reverse scan.

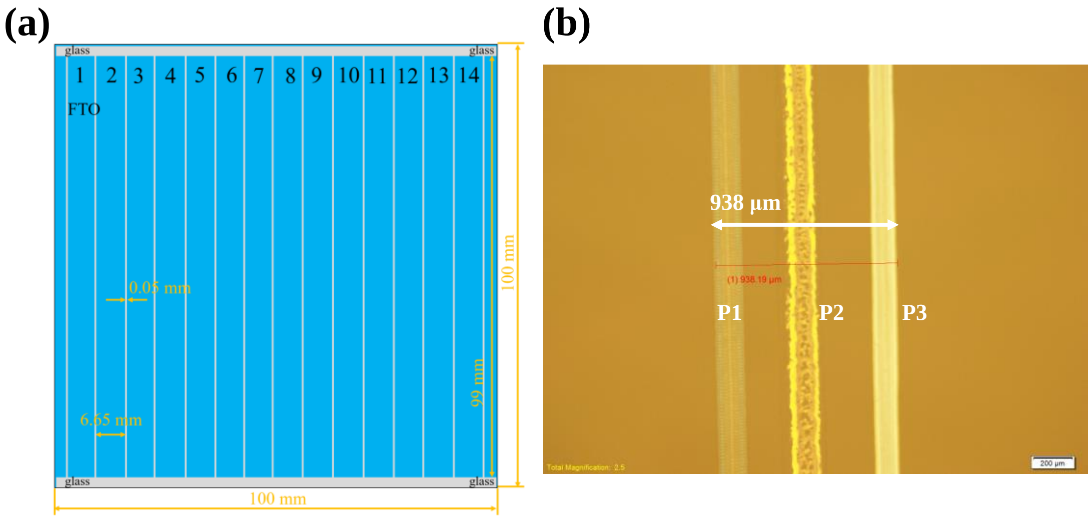

Fig. S21 (a) Top view of the 10 × 10 cm^2 FTO substrate pattern. (b) Optical photograph of the sub-cell separation including P1, P2 and P3 patterns in 10 × 10 cm^2 SnO_2/K-PSM. The corresponding GFF is determined to be approximately 0.860.

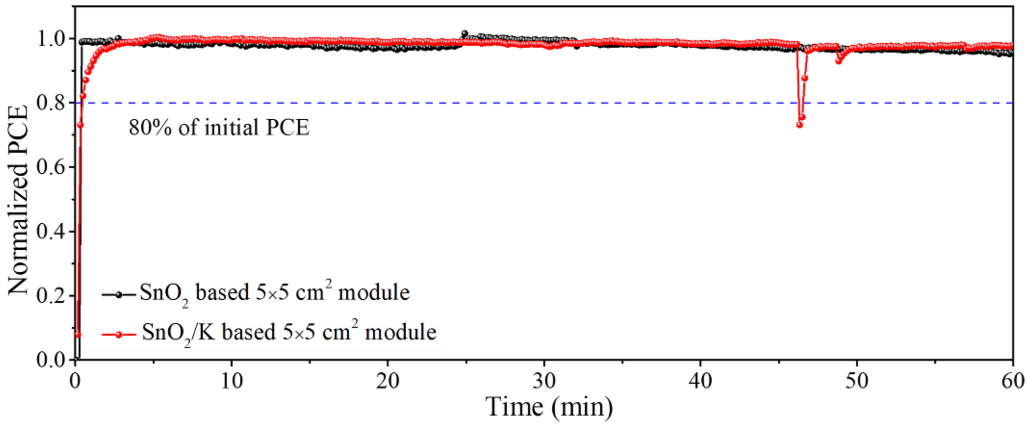

Fig. S22 Operational stability of the 5 × 5 cm^2 PSMs with encapsulation under a steady applied voltage and constant illumination (AM 1.5G, 100 mW cm^{-2}).

Tables

Table S1 The comparison of the efficiency and active area of perovskite solar cells by employing chemical bath deposition.

SnO_2 deposition	Device	Active area (cm^2)	Total area (cm^2)	PCE^a (%)	PCE^b (%)	Lifetime	Ref.
**CBD**	**FTO/SnO_2/PVSK/Spiro/Au**	**0.09**	**/**	**/**	**21.70**	**/**	**This work**
		**22.4**	**25 (5 × 5)**	**15.62**	**17.26**	**1006 h/T_{80}**	
		**91.8**	**100 (10 × 10)**	**11.80**	**13.72**	**/**	
CBD	FTO/SnO_2/PVSK/Spiro/Au	0.0937	/	/	25.4	/	[1]
		0.984	/	/	23	/	
CBD	FTO/SnO_2/PVSK/Spiro/Au	0.16	/	/	20.56	/	[2]
		20.0	36 (6 × 6)	/	15.76	/	
CBD	FTO/SnO_2/PCBM/PVSK/Spiro/Ag	0.0919	/	/	17.1	/	[3]
CBD	FTO/SnO_2/PVSK/Spiro/Au	0.16	/	/	20.7	/	[4]
CBD	FTO/SnO_2/PVSK/Spiro/Au	0.049	/	/	23.2	/	[5]
CBD	ITO/SnO_2/PVSK/Spiro/Au	0.1	/	/	14.8	/	[6]
Spin-coating+CBD	FTO/SnO_2/PVSK/SWNT-Spiro/Ag	0.0919	/	/	18.8	/	[7]

(Note: a is designated area of solar module; b is normalized by active area.)

Table S2 Fast and slow components for the TRPL decay.

Sample	A_1 (%)	τ_1 (ns)	A_2 (%)	τ_2 (ns)	$\tau_{average}$ (ns)
SnO_2	0.656	143.8	0.344	9.2	139.4
SnO_2/K	0.623	76.3	0.382	10.3	71.3

Table S3 Photovoltaics parameters of the SnO_2 based PSCs with different amounts of $KMnO_4$.

Sample	Scan direction	V_{oc} (V)	J_{sc} (mA cm^{-2})	FF	PCE (%)	HI
0 mM	FS.	1.069	22.82	0.699	17.05	1.18
	RS.	1.114	22.90	0.787	20.09	
5 mM	FS.	1.056	23.03	0.728	17.72	1.15
	RS.	1.096	23.06	0.807	20.39	
7 mM	FS.	1.107	23.28	0.778	20.07	1.06
	RS.	1.118	23.39	0.817	21.36	
8 mM	FS.	1.118	23.22	0.792	20.58	1.05
	RS.	1.127	23.39	0.823	21.70	
9 mM	FS.	1.108	23.08	0.786	20.10	1.03
	RS.	1.117	23.10	0.799	20.62	
10 mM	FS.	1.106	22.88	0.772	19.53	0.97
	RS.	1.111	22.61	0.757	19.03	

Table S4 Statistical photovoltaic parameters of open-circuit voltage (V_{oc}), short-circuit photocurrent density (J_{sc}), fill factor (FF) and power conversion efficiency (PCE) of the SnO_2-PSCs and SnO_2/K-PSCs based on 10 devices.

Sample	Scan direction	V_{oc} (V)	J_{sc} (mA cm^{-2})	FF	PCE (%)	HI
SnO_2	FS.	1.04±0.04	22.6±0.4	0.59±0.05	13.9±1.4	1.40
	RS.	1.09±0.02	22.6±0.4	0.79±0.01	19.5±0.5	
SnO_2/K	FS.	1.09±0.01	23.0±0.3	0.78±0.02	19.5±0.5	1.05
	RS.	1.11±0.01	23.1±0.3	0.81±0.01	20.6±0.5	

Table S5 Fitting parameters of the EIS measurement of the PSCs based on the SnO_2 and SnO_2/K substrates.

Sample	R_s (Ω)	R_{ct} (Ω)
SnO_2	26.01	62.57
SnO_2/K	16.67	22.44

Table S6 Statistical photovoltaic parameters of open-circuit voltage (V_{oc}), short-circuit photocurrent density (J_{sc}), fill factor (FF) and power conversion efficiency (PCE) of the SnO_2-PSCs and SnO_2/K-PSCs based on 10 devices after 5 months storage in ambient air in a dry room with a relative humidity of ~20% without any encapsulation

Sample	V_{oc} (V)	J_{sc} (mA cm^{-2})	FF	PCE (%)
SnO_2	1.08±0.02	21.9±0.5	0.73±0.03	17.4±0.8
SnO_2/K	1.11±0.01	22.4±0.3	0.76±0.04	18.8±0.8

Table S7 Photovoltaics parameters of 10 SnO_2 based perovskite solar modules (5 × 5 cm^2 PSMs) under reverse scan.

Sample	V_{oc} (V)	J_{sc} (mA cm^{-2})	FF	PCE (%)
1	7.158	2.72	0.582	11.34
2	6.560	2.59	0.677	11.48
3	6.920	2.82	0.632	12.36
4	6.186	2.86	0.647	11.44
5	7.663	2.87	0.620	13.64
6	7.635	2.56	0.530	10.35
7	7.656	2.60	0.580	11.55
8	6.311	2.83	0.677	12.09
9	7.218	2.58	0.583	10.87
10	7.770	2.89	0.649	14.58

Average	7.108±0.536	2.73±0.12	0.618±0.043	11.97±1.16

Table S8 Photovoltaics parameters of 10 SnO_2/K based perovskite solar modules (5 × 5 cm^2 PSMs) under reverse scan.

Sample	V_{oc} (V)	J_{sc} (mA cm^{-2})	FF	PCE (%)
1	7.520	2.62	0.706	13.91
2	7.602	2.84	0.674	14.55
3	6.738	2.91	0.698	13.68
4	7.400	2.76	0.631	12.88
5	7.437	2.55	0.674	12.76
6	6.573	2.81	0.672	12.41
7	6.735	2.79	0.725	13.63
8	7.478	2.80	0.661	13.83
9	7.450	2.62	0.664	12.93
10	7.591	2.95	0.699	15.62
Average	7.252±0.363	2.76±0.12	0.680±0.024	13.62±0.87

Table S9 Photovoltaics parameters of the champion efficiency of the SnO_2/K based perovskite solar modules (5 × 5 cm^2 PSMs).

Sample	Scan direction	V_{oc} (V)	J_{sc} (mA cm^{-2})	FF	PCE (%)	HI
SnO_2	FS.	7.413	2.66	0.379	7.49	1.95
	RS.	7.770	2.89	0.649	14.58	
SnO_2/K	FS.	7.166	2.98	0.667	14.25	1.10
	RS.	7.591	2.95	0.699	15.62	

Table S10 The comparison of the efficiency and operational stability of perovskite solar modules with the area over 20 cm^2.

Device	Active area (cm^2)	Total area (cm^2)	PCE[a] (%)	PCE[b] (%)	Lifetime	Ref
**FTO/SnO_2/$Cs_{0.05}FA_{0.85}MA_{0.10}PbI_{2.85}Br_{0.15}$/Spiro/Au**	**22.4**	**25 (5 × 5)**	**15.62**	**17.26**	**1006 h/T_{80}**	**This work**
	**91.8**	**100 (10 × 10)**	**11.80**	**13.72**	**/**	
FTO/SnO_2/$MAPbI_3$/Spiro/Au	21	36 (6 × 6)	/	18.13	100 h/T_{80}	[8]
ITO/P3HT/$MAPbI_3$/PCBM/Ag	36.6	49 (7 × 7)	/	16.06	250 h/T_{90}	[9]
ITO/PTAA/$MA_{0.7}FA_{0.3}PbI_3$/C_{60}/BCP/Cu	/	35.8	/	18.5	/	[10]
FTO/SnO_2/$[CsPbI_3]_{0.05}[(FAPbI_3)_{0.85}(MAPbBr_3)_{0.15}]_{0.95}$/Spiro/Au	25	36 (6.5 × 6.5)	15.3	16.02	/	[11]
FTO/TiO_2/$MAPbI_3$/Spiro/Au	50.6	100 (10 × 10)	/	12.6	1630 h/T_{80}	[12]
FTO/SnO_2/$(K_x(Cs_{0.05}(FA_{0.85}MA_{0.15})_{0.95}Pb(I_{0.85}Br_{0.15})_3)$/Spiro/Au	20	36 (6 × 6)	/	15.76	/	[2]
FTO/SnO_2/$(FAPbI_3)_{0.95}(CsPbBr_3)_{0.05}$/Spiro/Au	~20 (19.69)	25 (5 × 5)	/	17.94	/	[13]
ITO/SnO_2/$Cs_{0.05}FA_{0.54}MA_{0.41}Pb(I_{0.98}Br_{0.02})_3$/Spiro/Au	22.4	25 (5 × 5)	14.55	16.35	1625 h/T_{80}	[14]
FTO/ZnO-ZnS/$FA_{0.97}Cs_{0.03}PbI_3$/Spiro/Au	49	100 (10 × 10)	/	13.84	/	[15]
FTO/TiO_2/SnO_2/$Cs_{1-x}FA_xPb(I_yCl_{1-y})_3$/BJ-GO/TFB/Cr/Au	35.8	64 (8 × 8)	15.3	/	1000 h/T_{90}	[16]
ITO/SnO_2/EDTAK(EAMA)/$Cs_{0.05}FA_{0.54}MA_{0.41}Pb(I_{0.98}Br_{0.02})_3$/Spiro-P3HT/Au	22.4	25 (5 × 5)	16.6	18.2	2680 h/T_{80}	[17]
FTO/SnO_2/C_{60}/$Cs_{0.1}FA_{0.9}PbI_{2.9}Br_{0.1}$/Spiro/Au	22.4	25 (5 × 5)	~10	/	250 h/T_{90} 500 h/T_{80}	[18]
	91.8	100 (10 × 10)	9.34	10.37	/	
FTO/NiO_x/$[CH(NH_2)_2]_{0.85}[CH_3NH_3]_{0.15}Pb(I_{0.85}Br_{0.15})_3$/PCBM/BCP/Ag	36.1[ap]	64 (8 × 8)	/	15.6	1000 h/T_{91} 2222 h/T_{80}	[19]
FTO/SnO_2/$MAPbI_3$/Spiro/Au	22.8	25 (5 × 5)	12.03	/	280 h/T_{90} 515 h/T_{80}	[20]
FTO/TiO_2/$MAPbI_3$/Spiro/Au	36.1[ap]	64	/	15.7	500 h/T_{90}	[21]

		(8 × 8)			1000 h/T_{80}	
FTO/TiO_2/ZrO_2/(5-AVA)$_x$(MA)$_{1-x}$$PbI_3$/carbon	49	100 (10 × 10)	/	10.4	1000 h	[22]
FTO/c-TiO_2/ZrO_2/(5-AVA)$_x$(MA)$_{1-x}$$PbI_3$/carbon	46.7	100 (10 × 10)	/	11.2	> 10000 h	[23]
FTO/TiO_2/ZrO_2/(5-AVA)$_x$(MA)$_{1-x}$$PbI_3$/carbon	31	50 (5 × 10)	/	10.46	72 h	[24]
	70	100 (10 × 10)	/	10.75	/	

(Note: a is designated area of solar module; b is normalized by active area.)

Table S11 Photovoltaics parameters of 10 SnO_2/K based perovskite solar modules (10 × 10 cm^2 PSMs) under reverse scan.

**Sample**	**V_{oc} (V)**	**J_{sc} (mA cm^{-2})**	**FF**	**PCE (%)**
1	12.337	1.38	0.586	9.97
2	12.615	1.21	0.634	9.68
3	13.698	1.33	0.571	10.39
4	11.713	1.27	0.539	7.99
5	12.880	1.39	0.611	10.92
6	12.828	1.38	0.588	10.45
7	13.498	1.31	0.510	8.99
8	12.346	1.20	0.636	9.45
9	12.661	1.36	0.637	10.98
10	13.158	1.38	0.648	11.80
Average	12.773±0.529	1.32±0.07	0.596±0.042	10.06±0.99

References

1. J.J. Yoo, G. Seo, M.R. Chua, T.G. Park, Y. Lu et al., Efficient perovskite solar cells via improved carrier management. Nature **590**, 587-593 (2021). https://doi.org/10.1038/s41586-021-03285-w
2. T. Bu, X. Liu, Y. Zhou, J. Yi, X. Huang et al., A novel quadruple-cation absorber for universal hysteresis elimination for high efficiency and stable perovskite solar cells. Energy Environ. Sci. **10**(12), 2509-2515 (2017). https://doi.org/10.1039/C7EE02634J
3. D. P. McMeekin, G. Sadoughi, W. Rehman, G. E. Eperon, M.l Saliba et al., A mixed-cation lead mixed-halide perovskite absorber for tandem solar cells. Science **351**(6269), 151-155 (2016). https://doi.org/10.1126/science.aad5845
4. E.H. Anaraki, A. Kermanpur, L. Steier, K. Domanski, T. Matsui et al., Highly efficient and stable planar perovskite solar cells by solution-processed tin oxide. Energy Environ. Sci. **9**(10), 3128-3134 (2016). https://doi.org/10.1039/C6EE02390H
5. E.H. Jung, B. Chen, K. Bertens, M. Vafaie, S. Teale et al., Bifunctional surface engineering on SnO_2 reduces energy loss in perovskite solar cells. ACS Energy Lett. **5**(9), 2796-2801 (2020). https://doi.org/10.1021/acsenergylett.0c01566
6. J. Barbe, M.L. Tietze, M. Neophytou, B. Murali, E. Alarousu et al., Amorphous tin oxide as a low-temperature-processed electron-transport layer for organic and hybrid perovskite solar cells. ACS Appl. Mater. Interfaces **9**(13), 11828-11836 (2017). https://doi.org/10.1021/acsami.6b13675
7. S.N. Habisreutinger, B. Wenger, H.J. Snaith, R.J. Nicholas, Dopant-free planar n–i–p perovskite solar cells with steady-state efficiencies exceeding 18%. ACS Energy Lett. **2**(3), 622-628 (2017). https://doi.org/10.1021/acsenergylett.7b00028
8. J. Li, H. Wang, X.Y. Chin, H.A. Dewi, K. Vergeer et al., Highly efficient thermally co-evaporated perovskite solar cells and mini-modules, Joule **4**(5), 1035-1053 (2020). https://doi.org/10.1016/j.joule.2020.03.005
9. H.-H. Huang, Q.-H. Liu, H. Tsai, S. Shrestha, L.-Y. Su et al., A simple one-step method with wide processing window for high-quality perovskite mini-module

fabrication. Joule (2021). https://doi.org/10.1016/j.joule.2021.02.012

10. S. Chen, X. Xiao, H. Gu, J. Huang, Iodine reduction for reproducible and high-performance perovskite solar cells and modules. Sci. Adv. **7**(10), eabe8130 (2021). https://doi.org/10.1126/sciadv.abe8130
11. G.S. Han, J. Kim, S. Bae, S. Han, Y.J. Kim et al., Spin-coating process for 10 cm × 10 cm perovskite solar modules enabled by self-assembly of SnO_2 nanocolloids. ACS Energy Lett. **4**(8), 1845-1851 (2019). https://doi.org/10.1021/acsenergylett.9b00953
12. A. Agresti, S. Pescetelli, A.L. Palma, A.E. Del Rio Castillo, D. Konios et al., Graphene interface engineering for perovskite solar modules: 12.6% power conversion efficiency over 50 cm^2 active area. ACS Energy Lett. **2**(1), 279-287 (2017). https://doi.org/10.1021/acsenergylett.6b00672
13. D.-K. Lee, K.-S. Lim, J.-W. Lee, N.-G. Park, Scalable perovskite coating via anti-solvent-free Lewis acid–base adduct engineering for efficient perovskite solar modules. J. Mater. Chem. A **9**(5), 3018-3028 (2021). https://doi.org/10.1039/D0TA10366G
14. G. Tong, D.-Y. Son, L. K. Ono, Y. Liu, Y. Hu et al., Scalable fabrication of > 90 cm^2 perovskite solar modules with > 1000 h operational stability based on the intermediate phase strategy. Adv. Energy Mater. **11**(10), 2003712 (2021). https://doi.org/10.1002/aenm.202003712
15. R. Chen, Y. Wu, Y. Wang, R. Xu, R. He et al., Crown ether-assisted growth and scaling up of $FACsPbI_3$ films for efficient and stable perovskite solar modules. Adv. Funct. Mater. **31**(11), 2008760 (2021). https://doi.org/10.1002/adfm.202008760
16. Y. Sha, E. Bi, Y. Zhang, P. Ru, W. Kong et al., A scalable integrated dopant - free heterostructure to stabilize perovskite solar cell modules. Adv. Energy Mater. **11**(5), (2021) 2003301. https://doi.org/10.1002/aenm.202003301
17. Z. Liu, L. Qiu, L.K. Ono, S. He, Z. Hu et al., A holistic approach to interface stabilization for efficient perovskite solar modules with over 2,000-hour operational stability. Nat. Energy **5**, 596-604 (2020). https://doi.org/10.1038/s41560-020-0653-2

18. L. Qiu, S. He, Y. Jiang, D.-Y. Son, L.K. Ono et al., Hybrid chemical vapor deposition enables scalable and stable Cs-FA mixed cation perovskite solar modules with a designated area of 91.8 cm^2 approaching 10% efficiency. J. Mater. Chem. A **7**(12), 6920-6929 (2019). https://doi.org/10.1039/C9TA00239A
19. E. Bi, W. Tang, H. Chen, Y. Wang, J. Barbaud et al., Efficient perovskite solar cell modules with high stability enabled by iodide diffusion barriers. Joule **3**(11), 2748-2760 (2019). https://doi.org/10.1016/j.joule.2019.07.030
20. L. Qiu, Z. Liu, L.K. Ono, Y. Jiang, D.Y. Son et al., Scalable fabrication of stable high efficiency perovskite solar cells and modules utilizing room temperature sputtered SnO_2 electron transport layer. Adv. Funct. Mater. **29**(47), 1806779 (2018). https://doi.org/10.1002/adfm.201806779
21. H. Chen, F. Ye, W. Tang, J. He, M. Yin et al., A solvent- and vacuum-free route to large-area perovskite films for efficient solar modules. Nature **550**, 92-95 (2017). https://doi.org/10.1038/nature23877
22. Y. Hu, S. Si, A. Mei, Y. Rong, H. Liu et al., Stable large-area (10 × 10 cm^2) printable mesoscopic perovskite module exceeding 10% efficiency. Sol. RRL **1**(2), 1600019 (2017). https://doi.org/10.1002/solr.201600019
23. G. Grancini, C. Roldan-Carmona, I. Zimmermann, E. Mosconi, X. Lee et al., One-Year stable perovskite solar cells by 2D/3D interface engineering. Nat. Commun. **8**, 15684 (2017). https://doi.org/10.1038/ncomms15684
24. A. Priyadarshi, L.J. Haur, P. Murray, D. Fu, S. Kulkarni et al., A large area (70 cm^2) monolithic perovskite solar module with a high efficiency and stability. Energy Environ. Sci. **9**(12), 3687-3692 (2016). https://doi.org/10.1039/C6EE02693A
